# Supplementary material for: A Retinal Circuit Generating a Dynamic Predictive Code for Oriented Features
Source: Neuron. 2019 Jun 19;102(6):1211–1222.e3. doi: 10.1016/j.neuron.2019.04.002 (PMC6591004; doi:10.1016/j.neuron.2019.04.002)
Supplement: Document S1. Figures S1–S5 [file mmc1.pdf]

Neuron, Volume *102*

Supplemental Information

A Retinal Circuit Generating

a Dynamic Predictive Code for Oriented Features

Jamie Johnston, Sofie-Helene Seibel, Léa Simone Adele Darnet, Sabine Renninger, Michael Orger, and Leon Lagnado

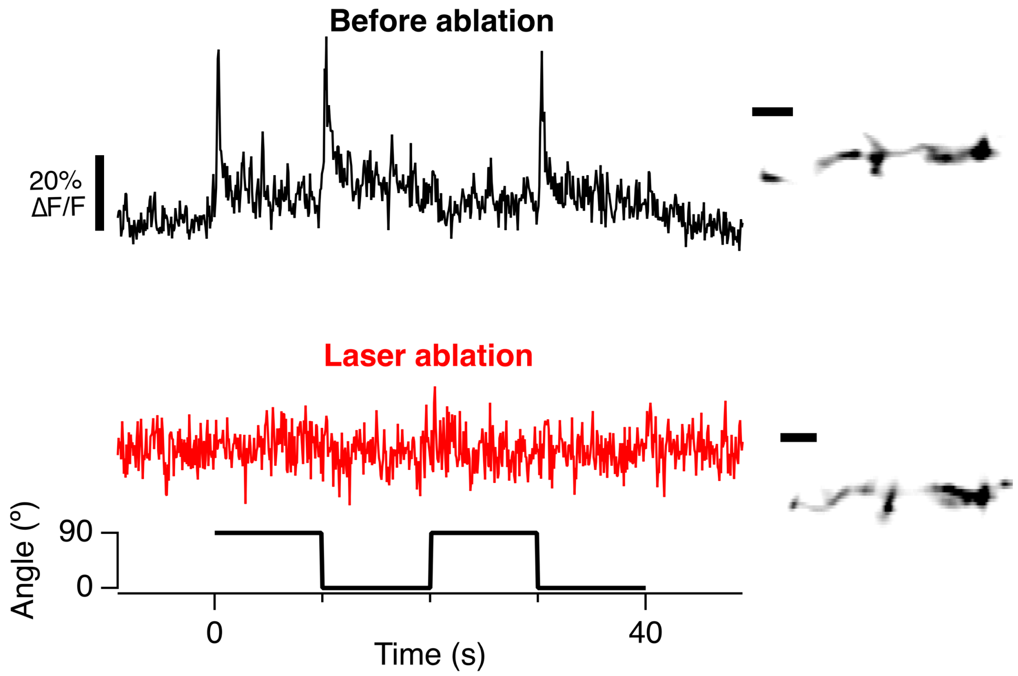

Figure S1. iGluSnFR signals were abolished after laser ablation of the soma, related to Figure 1. The iGluSnFR signal from an RGC axon terminal in response to a full-field grating reversing contrast at 5 Hz that switched orientation from 90º to 0º at 10 s intervals. This RGC was statically tuned to the vertical. The black and red traces show the responses before and after laser ablation of the RGC's cell body. The axonal arborization in the tectum is shown to the right. Scale bar 5 μm. This behaviour was observed in all three RGCs tested in this way, indicating that iGluSnFR signals measured in the axon of RGCs did not reflect glutamate spillover.

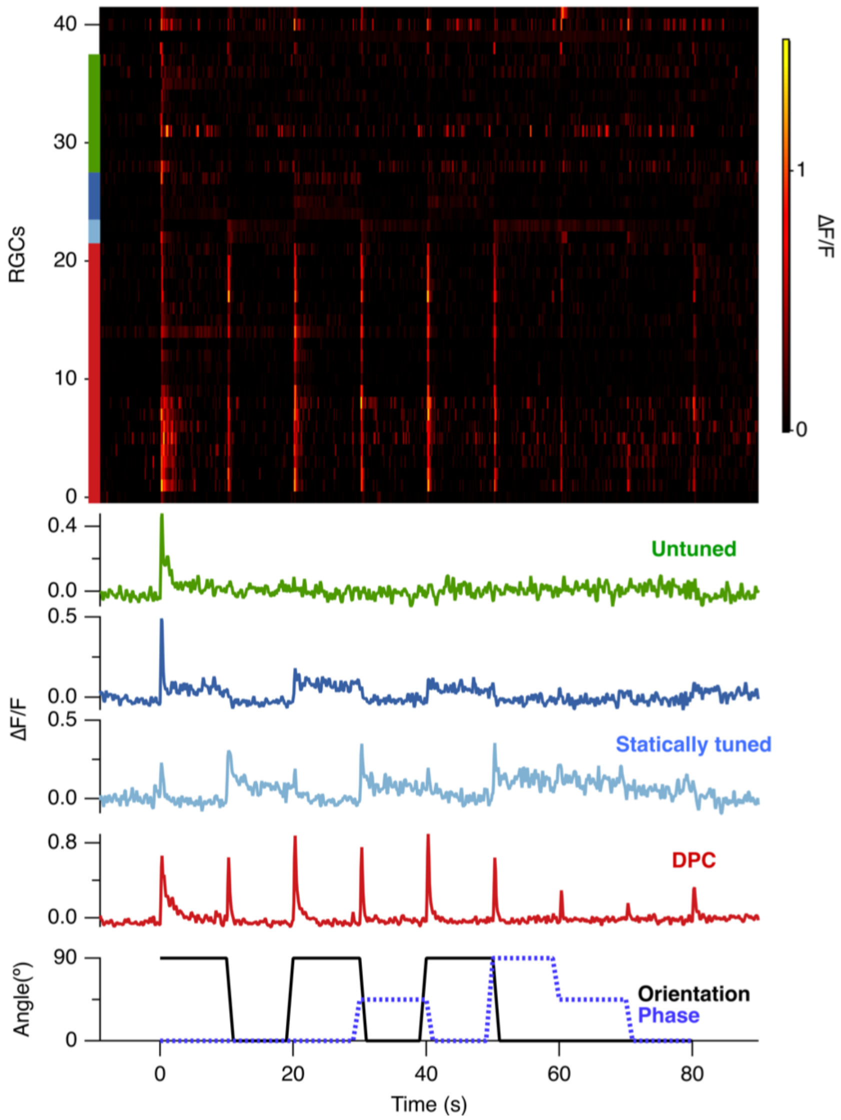

Figure S2. Responses to changes in orientation were not caused by changes in local luminance, related to Figure 1. Raster plot of the responses of 42 retinal ganglion cell terminals to a full-field grating reversing contrast at 5 Hz that switched orientation from 90° to 0° at 10 s intervals. Shifts in orientation were applied at three different phases of the grating (0°, 45° and 90°) and then phase changes alone (90° -> 45° and 45° -> 0°) were applied at a constant orientation of 0°. The traces below are the average responses for neurons classified as either untuned to orientation (green), statically tuned (light and dark blue) or those exhibiting dynamic predictive coding (red). Note that the 22 DPC neurons generated very similar responses to changes in orientation at different phases of the grating and that changes in phase alone generated significantly smaller responses.

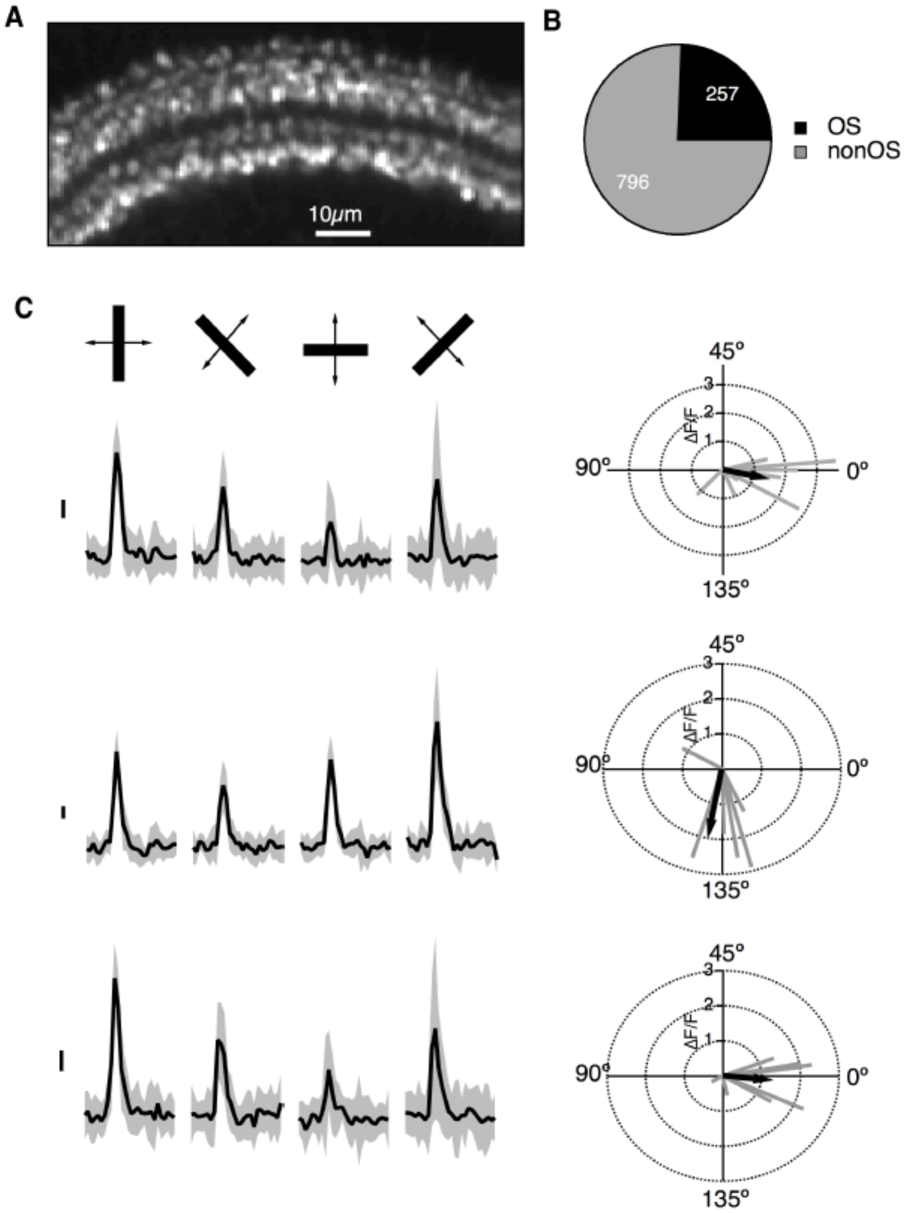

Figure S3. Orientation selectivity in bipolar cell synapses measured with moving bars, related to Figure 3. A) An array of bipolar cell terminals labelled with SyGCaMP6f imaged *in vivo*. **B)** 25% of 1053 measured synapses displayed a significant orientation preference with a false positive rate of 1%. The distribution of orientation preferences for these terminals are shown in Fig. 3F. **C)** *Left:* Example responses from 3 terminals to bars of different orientations moving across the field of view mean (black) ± SD (grey) of 10 repetitions. *Right:* The vector sum of each of the 10 trials plotted in orientation space (grey) with the average vector sum shown in black. To detect whether an individual terminal displayed a significant orientation preference we performed Moore's version of Rayleigh's test on the distribution of vector sums.

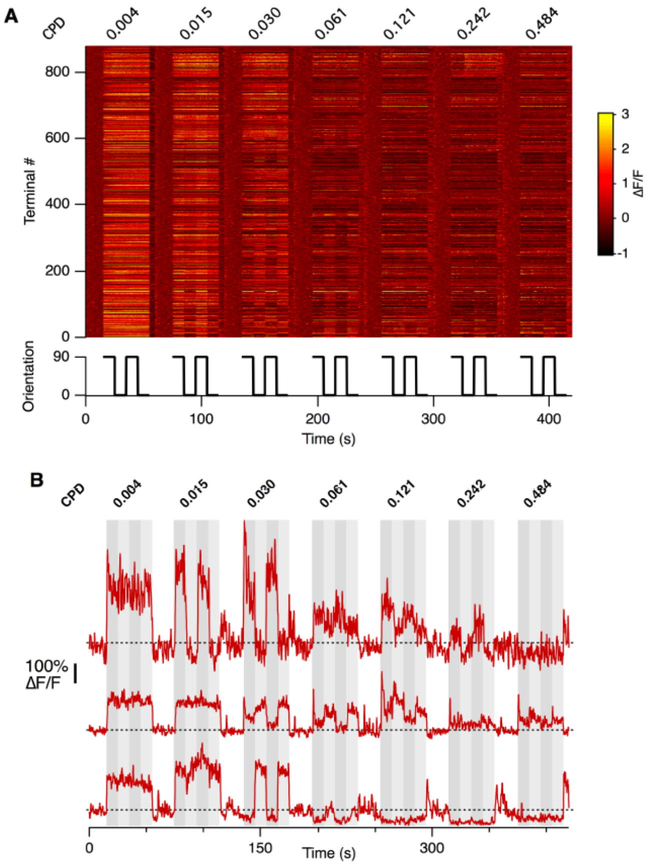

Figure S4. Spatial frequency tuning of bipolar cell synapses measured with SyGCaMP6f, related to Figure 3. A) The responses of 819 retinal bipolar cell synapses to gratings of spatial frequency ranging from 0.004 cycles per degree (CPD) to 0.484 CPD, 0.004 CPD is equivalent to full field. Each grating was given in a pseudo random order and then de-shuffled and concatenated for display purposes. This data generated the spatial frequency tuning shown in Fig. 3D. **B)** Examples from 3 bipolar terminals to gratings of varying spatial frequency.

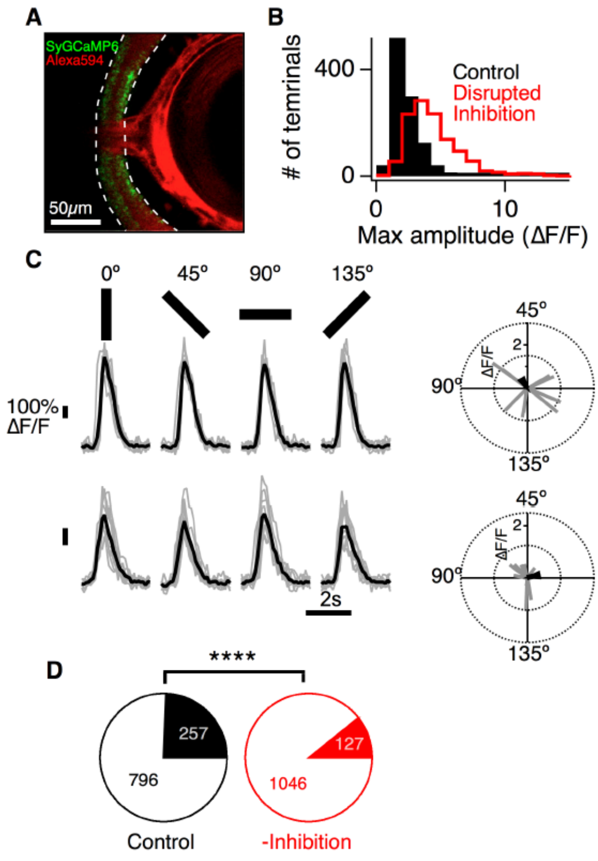

Figure S5. Inhibition contributes to the orientation sensitivity of bipolar cell terminals, related to Figure 3. A) Wide field of view of a zebrafish eye after an intravitreal injection of ~4 nl of a solution containing 10 mM strychnine, 10 mM gabazine and 1 mM Alexa 594. The inner plexiform layer is demarcated with dashed white lines with SyGCaMP6 expressing bipolar cell terminals labelled in green. The Alexa 594 signal is concentrated in the vitreous chamber and can be detected in the inner plexiform layer. **B)** Histograms comparing the average peak amplitudes of the response to the preferred stimuli for control (black, n=1053 terminals) and with intravitreal injection of inhibitory antagonists (red, n=1173, 7 fish). With inhibition blocked the responses of bipolar terminals tended to be larger (P<0.0001, Mann-Whitney test). **C)** Example responses from 2 terminals to bars of different orientations moving across the field of view, recorded in a retina with inhibitory antagonists blocked. Grey traces represent the 10 individual trials with the average shown in black. *Right:* The vector sum of each of the 10 trails plotted in orientation space (grey) with the average vector sum shown in black. **D)** The proportion of OS terminals was significantly lower in retinae where inhibition was blocked (red) when compared to control (black), P<0.0001, Fisher's exact test).
