# Supplementary material for: A Retinal Circuit Generating a Dynamic Predictive Code for Oriented Features
Source: Neuron. 2019 Jun 19;102(6):1211–1222.e3. doi: 10.1016/j.neuron.2019.04.002 (PMC6591004; doi:10.1016/j.neuron.2019.04.002)
Supplement: Document S2. Article plus Supplemental Information [file mmc2.pdf]

Article

Neuron

A Retinal Circuit Generating a Dynamic Predictive Code for Oriented Features

Highlights

- A dynamic predictive code (DPC) for oriented features is generated in the retina
- Orientation is first computed in synapses of bipolar cells via lateral inhibition
- Synapses coding different orientations converge on an RGC, generating a DPC
- Feedforward inhibition onto RGCs limits their activation to a narrow time window

Authors

Jamie Johnston, Sofie-Helene Seibel, Léa Simone Adele Darnet, Sabine Renninger, Michael Orger, Leon Lagnado

Correspondence

l.lagnado@sussex.ac.uk

In Brief

Predictive coding prevents transmission of redundant information by sensory systems. Johnston et al. show how this computation is implemented and adjusted by the neural circuitry of the retina as it processes signals representing the orientation of visual features.

Johnston et al., 2019, Neuron *102*, 1211–1222
June 19, 2019 Crown Copyright © 2019 Published by Elsevier Inc.
https://doi.org/10.1016/j.neuron.2019.04.002

CellPress

CellPress

A Retinal Circuit Generating a Dynamic Predictive Code for Oriented Features

Jamie Johnston,[2] Sofie-Helene Seibel,[1] Léa Simone Adele Darnet,[1] Sabine Renninger,[3] Michael Orger,[3] and Leon Lagnado[1,4,*]
[1]Sussex Neuroscience, School of Life Sciences, University of Sussex, Brighton BN1 9QG, UK
[2]School of Biomedical Sciences, Faculty of Biological Sciences, University of Leeds, Leeds LS2 9JT, UK
[3]Champalimaud Centre for the Unknown, Lisbon 1400-038, Portugal
[4]Lead Contact
*Correspondence: l.lagnado@sussex.ac.uk
https://doi.org/10.1016/j.neuron.2019.04.002

SUMMARY

Sensory systems must reduce the transmission of redundant information to function efficiently. One strategy is to continuously adjust the sensitivity of neurons to suppress responses to common features of the input while enhancing responses to new ones. Here we image the excitatory synaptic inputs and outputs of retinal ganglion cells to understand how such dynamic predictive coding is implemented in the analysis of spatial patterns. Synapses of bipolar cells become tuned to orientation through presynaptic inhibition, generating lateral antagonism in the orientation domain. Individual ganglion cells receive excitatory synapses tuned to different orientations, but feedforward inhibition generates a high-pass filter that only transmits the initial activation of these inputs, removing redundancy. These results demonstrate how a dynamic predictive code can be implemented by circuit motifs common to many parts of the brain.

INTRODUCTION

A general principle in understanding the design of sensory systems is the need to encode information efficiently which, in turn, requires removal of redundancies in the signal received from the outside world (Barlow, 1961; Fairhall et al., 2001; Srinivasan et al., 1982; Sterling and Laughlin, 2015). This principle helps us understand why the retina does not operate simply like a camera conveying a stream of intensity values for each pixel. Natural images contain a large amount of redundant information because pixels nearby in space and/or time tend to be correlated (Atick and Redlich, 1992; Masland, 2012; van Hateren, 1992). Rather than continuously transmitting the presence of an unchanging visual input, many retinal neurons preferentially signal deviations from the local image statistics, such as regions of contrast or the sudden movement of an object. Such computations are direct reflections of spatial receptive fields with antagonistic surrounds and temporal receptive fields signaling changes in light intensity. Representing information by ignoring statistical regularities to highlight unusual components is termed predictive coding, an approach widely used in informatics to, for instance, compress images (Kingston and Autrusseau, 2008). Examples of predictive coding have been identified throughout the brain, including the circuits involved in vision (Ekman et al., 2017; Hosoya et al., 2005; Nirenberg et al., 2010; Sharpee et al., 2006), hearing (Smith and Lewicki, 2006; Sohoglu and Chait, 2016), and touch (Peyrache et al., 2015), encoding of reward in the midbrain (Parker et al., 2016; Schultz, 2016) and of space in the hippocampus and entorhinal cortex (Hindy et al., 2016; Stachenfeld et al., 2017). Although a number of models have been proposed for the neural implementation of predictive codes (Bastos et al., 2012; Hosoya et al., 2005; Rao and Ballard, 1999), the circuit mechanisms have not been clearly identified.

Predictive coding can be a dynamic process, consistent with the animal's need to adjust to sensory environments with different statistics (Sharpee et al., 2006; Smith and Lewicki, 2006). For instance, the retina can adjust within seconds to changes in the variance in light intensity (contrast), and this occurs through changes in the strength of both excitatory and inhibitory synapses (Kastner and Baccus, 2011; Nikolaev et al., 2013; Kastner and Baccus, 2014). Another example of adaptation occurs in response to changes in spatial correlations within the visual input generated by objects of different size and orientation (Benucci et al., 2013; Dragoi et al., 2000; Müller et al., 1999). Neurons that signal and adapt to orientation are observed in the retina (Nath and Schwartz, 2017; Venkataramani and Taylor, 2016) and visual cortex (Hubel and Wiesel, 1959; Iacaruso et al., 2017; Kondo and Ohki, 2016; Ohki et al., 2005). Further, some retinal ganglion cells (RGCs) implement a dynamic predictive code (DPC) by rapidly altering their sensitivity to orientation in response to changes in the distribution of spatial correlations, becoming less sensitive to orientations that are common features of the input and more sensitive to orientations that are uncommon (Gollisch and Meister, 2010; Hosoya et al., 2005; Lesica et al., 2007).

The neural circuitry by which the visual system implements a DPC is not understood, either in the retina (Kastner and Baccus, 2014) or in the cortex (Benucci et al., 2013; Rao and Ballard, 1999). One model proposes construction of a modifiable pattern detector that is fed by an array of excitatory subunits, each one tuned to a different stimulus pattern; when one of these detector subunits is driven strongly, it depresses and makes a smaller

This is an open access article under the CC BY license (http://creativecommons.org/licenses/by/4.0/).

contribution to the activity of the output neuron, which therefore becomes more sensitive to other, rarer, patterns (Bastos et al., 2012; Gollisch and Meister, 2010; Graham, 2001; Hosoya et al., 2005). This model is attractive but is not thought to operate in the retina because bipolar cells providing excitatory inputs to RGCs do not appear to be sensitive to orientation (Hosoya et al., 2005). An alternative hypothesis of "network plasticity" has therefore been proposed, in which the locus of the adaptive changes are the synapses RGCs receive from the inhibitory amacrine cells, with these synapses obeying an anti-Hebbian plasticity rule that strengthens them when they are co-activated with the RGC (Hosoya et al., 2005). In this scheme, a vertically orientated stimulus will strengthen inhibitory inputs above and below the RGC, making it more sensitive to horizontal orientations.

To identify the circuit mechanisms generating dynamic predictive coding of spatial patterns, we used an optical approach based on the fluorescent glutamate sensor iGluSnFR (Marvin et al., 2013) to compare the synaptic output from individual RGCs with the excitatory synaptic inputs they receive from an array of bipolar cells. We probed these synapses using gratings of different orientations and found that many are strongly tuned to orientation through lateral antagonism in the orientation domain. Further, a subset of RGCs was driven by a mixture of excitatory synapses tuned to different orientations, providing the basic connectivity for a modifiable pattern detector. The nulling of a constant input is then generated through at least two mechanisms: depression of excitatory synapses and fast feedforward inhibition (FFI). The basic features of this circuit are found in many other parts of the brain and may provide a general design for the dynamic tuning of neurons and removal of redundancy.

RESULTS

Imaging the Input and Output from Individual Retinal Ganglion Cells

The signals RGCs deliver to the brain depend on the integration of a variety of synaptic inputs, often with mixed properties. Understanding how the activity of these inputs determines the output of the neuron has been difficult because individual synapses on a dendrite cannot be isolated using electrophysiology (Branco and Häusser, 2011; Schmidt-Hieber et al., 2017). To overcome this problem, we used *in vivo* imaging in larval zebrafish expressing iGluSnFR over the surface of subsets of RGCs (Figure 1A). Visual stimuli generated "hotspots" of iGluSnFR fluorescence on dendrites (Figure 1B), and these displayed many of the functional properties expected of glutamate release from the synapses of bipolar cells, such as adaptation to contrast (Marvin et al., 2013; Figure 2). The axons of these same RGCs could then be tracked to monitor the signal delivered in the optic tectum, also by release of glutamate (Figure 1A). iGluSnFR signals in the tectum were abolished when the cell body in the retina was ablated, demonstrating that they reflected the synaptic release of glutamate from the imaged neuron rather than spillover from neighboring cells (Figure S1).

We explored changes in tuning to orientation by presenting gratings that continuously reversed contrast at 5 Hz and then switching the orientation from horizontal (90°) to vertical (0°) every 5–10 s. In 144 of 148 RGCs, the first presentation of the grating elicited a strong and transient output simply because of the increase in contrast (Figure 1C; Figure S2). Subsequent shifts in orientation did not elicit a response in 39 of the cells (Figure 1F), but in 107 RGCs it did (Nikolaou et al., 2012). For instance, the upper red trace in Figure 1E shows a neuron generating a strong response to the vertical grating (R_0) but very weak responses to the horizontal grating alone (R_{90}), whereas the lower red trace is a neuron that responds to the horizontal but not the vertical. Of the RGCs whose response varied with orientation, 61 exhibited relatively static tuning, responding more strongly to one orientation and adapting incompletely or not at all (Figure 1E, red traces). These statically tuned RGCs had an orientation-selective index (OSI), defined as $|R_0\text{-}R_{90}|/(|R_0|+|R_{90}|)$, that averaged 0.69 (Figure 1D). The remaining 46 RGCs were qualitatively different in generating a transient glutamatergic output at both the horizontal-to-vertical and vertical-to-horizontal transitions, and three examples are shown by the black traces in Figure 1E.

RGCs Dynamically Tuned to Different Orientations

Are RGCs transmitting a signal to the tectum at each change in stimulus orientation becoming retuned, or might they simply be responding to local changes in luminance when the grating is rotated within their receptive field? To distinguish between these possibilities, we carried out two tests. First, responses to changes in orientation were measured at two spatial phases of the grating shifted by 45°, and then these were compared with responses to changes in phase (steps of 45°) at a fixed orientation. The example traces in Figure 1E show that recovery of sensitivity to the orthogonal stimulus was very similar at different phases and that a phase change alone usually elicited no response, or else a much smaller response, than the orthogonal stimulus (dashed blue box). The averaged synaptic output to this protocol across all 42 RGCs tested in this way is provided in Figure S2. Again, a subset of RGCs responded to orthogonal stimuli irrespective of phase and in these cells phase changes alone elicited much smaller responses. These results confirm that RGCs responding to all changes in stimulus orientation were dynamically altering their tuning to this feature of the stimulus.

Among the 46 RGCs that were dynamically tuned, the response to a shift in orientation depressed by 92.0% ± 0.1% within 0.26 ± 0.31 s, effectively suppressing the transmission of redundant information. Within ~9 s of this profound adaptation, the neurons had become re-tuned to allow signaling of a future change in stimulus orientation, demonstrating the ability to generate a dynamic predictive code (Hosoya et al., 2005; Sharpee et al., 2006; Smith and Lewicki, 2006). Together, the results in Figure 1 demonstrate that the retina of zebrafish informs the brain of orientated features in the visual world through two functionally distinct groups of RGCs, either dynamically or statically tuned.

Detection of Spatial Patterns Originates in the Synaptic Compartment of Bipolar Cells

To investigate how some RGCs dynamically alter their tuning to spatial patterns, we began by asking whether their excitatory inputs might be sensitive to orientation. Electrical recordings in the somata of bipolar cells have not yet revealed any orientation selectivity (Hosoya et al., 2005), but it is known that inhibitory

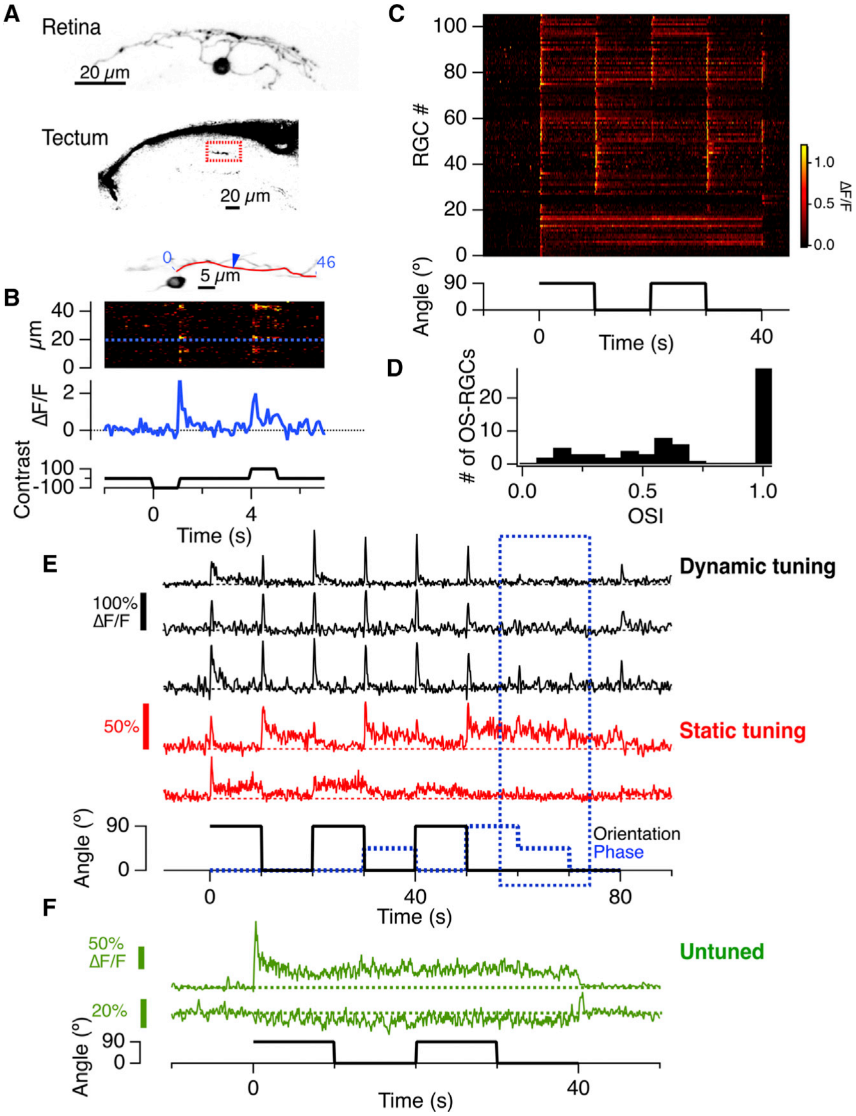

Figure 1. Imaging the Input and Output from Individual Retinal Ganglion Cells

(A) Maximum intensity projection of an RGC labeled with iGluSnFR imaged *in vivo* and its axon terminal in the optic tectum.

(B) Top: a single plane imaged through an RGC. Center: a raster plot showing the time series of the iGluSnFR signal along a dendrite (red line) in response to changes in full-field luminance. Bottom: time course of the iGluSnFR signal at the point indicated by the blue arrow (corresponding to the dashed blue line above).

(C) Raster plot of the responses of 106 retinal ganglion cell terminals to a full-field grating reversing contrast- at 5 Hz. The grating appears at time zero at an orientation of 90° and then switches orientation between 90° and 0° at 10 s intervals. Neurons 0–27 only responded to changes in contrast. Neurons exhibiting a dynamic predictive code (72–96) responded with transient increases in glutamate at each change in stimulus orientation.

(D) Histogram of the OSI for the 66 RGCs in (D) showing significant orientation selectivity.

(E) Top (black): examples of output from three RGCs displaying DPC. The black trace shows changes in orientation of the grating, which were applied at three different spatial phases of the grating (0°, 45°, and 90°) shown by the dashed blue trace. Note that responses to a change in orientation were similar at all phases, while a change in phase alone generated a small response in just one of the three neurons (blue dashed box). Bottom (red): examples of output from two RGCs with static tuning to orientation.

(F) Examples of output from two RGCs insensitive to orientation: the lowermost was inhibited by an increase in contrast.

inputs to the synaptic compartment can dramatically modify the electrical signal that drives their output (Asari and Meister, 2012; Nikolaev et al., 2013). By imaging iGluSnFR, we directly assessed the signal transmitted by bipolar cells. The raster plot in Figure 2A shows iGluSnFR signals from 606 synaptic inputs onto the dendrites of 27 RGCs; 47% of these synapses displayed significant orientation selectivity and individual examples are shown in Figure 2B. The distribution of OSIs measured for bipolar cell synapses displayed two distinct populations; the smallest group (30%) had a median OSI of 0.3, but the remaining 70% were almost perfectly selective for one orientation over the orthogonal (Figure 2C). We confirmed this finding by surveying the whole population of bipolar cell synapses using zebrafish expressing a fast version of GCaMP6 fused to synaptopyhsin (Johnston et al., 2014); ~25% of these displayed orientation selectivity when tested with either moving bars (Figure S3) or the grating stimulus (Figure S4), and there was a clear preference for vertically oriented stimuli (Figure 3F). These results demonstrate that the analysis of orientation within the zebrafish visual system begins within the synaptic compartment of bipolar cells.

The kinetics of the signals encoding orientation within the retina and the optic tectum were profoundly different. Figure 2D compares the averaged output of RGCs displaying dynamic predictive coding (black) with the subset of bipolar cell synapses tuned to the horizontal (red). Bipolar cell outputs declined by an average of 49% over a 10 s period, in general agreement with the kinetics of contrast adaptation at the bipolar cell synapse measured using electrophysiology in slices (Jarsky et al., 2011) or the optical reporter sypHy *in vivo* (Nikolaev et al., 2013; Odermatt et al., 2012). In contrast, the output of RGCs adapted completely with a dominant time constant of between 0.17 s and 0.25 s. Although depression intrinsic to bipolar cell synapses will also contribute to nulling the output to a constant signal (Gollisch and Meister, 2010), this process is too slow and incomplete to account for the extent of adaptation in the signal delivered to the tectum (Figure 2D). The rapid "zeroing" of the retinal output in the face of an unchanging visual input is key to removing redundancy in the signal transmitted to the brain, and these results demonstrate that the retina carries out this operation *after* the bipolar cell synapse.

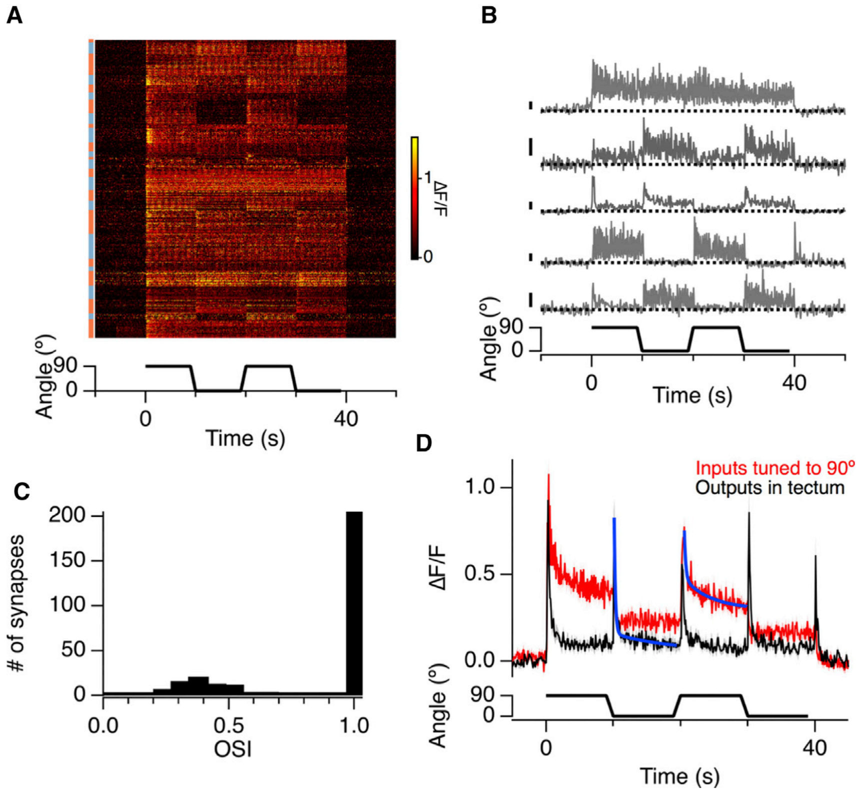

Figure 2. The Output from Individual Bipolar Cells Displays Orientation Selectivity but Not Dynamic Predictive Coding

(A) Raster plot showing the iGluSnFR signal of 606 bipolar cell inputs to 27 retinal ganglion cells in response to a full-field grating that switched orientation from 90° to 0° (stimulus as in Figure 1). The alternating blue and orange bars on the left indicate different RGCs. None of the synapses were activated at each change in orientation.

(B) Activity at five example synapses on RGC dendrites. The first is not sensitive to orientation, whereas the lower four were stably tuned, preferring either the vertical or horizontal. All scale bars, 50% ΔF/F.

(C) Histogram of the OSIs for the orientation-selective inputs measured in (A). Note the two distinct populations.

(D) A comparison of the glutamatergic output of bipolar cell synapses tuned to 90° (red, n = 151) and RGCs displaying dynamic predictive coding (black, n = 23). Shaded areas show ± SEM. Note the differences in the time course of adaptation. Solid blue lines are double exponential fits of the form $y = y_o + A1((t - t_o)/t_1) + A_2((t - t_o)/t_2)$. The time constants of depression for the bipolar synapses were 0.21 s and 4.2 s and accounted for 61% and 39% of the decay, respectively. In contrast, the output of RGCs in the tectum was dominated by a fast process of adaptation in which 90% of the decay occured with a time constant of 0.18 s.

Intrinsic and Extrinsic Mechanisms Generating Orientation Tuning in Bipolar Cell Synapses

How do bipolar cell outputs become tuned to orientation? Two potential mechanisms that might operate together are shown in Figure 3A. The first involves synapses with asymmetric receptive field centers causing features aligned with the longer axis to generate larger responses than those at other angles. We investigated this possibility by measuring calcium signals within bipolar cell terminals using SyGCaMP6f and mapping their receptive fields using the technique of filtered back projection (Johnston et al., 2014). Consistent with the dendritic field shapes of zebrafish bipolar cells (Li et al., 2012), the large majority of terminals had receptive fields displaying some degree of ellipticity (Figure 3B). The median ellipticity was 0.30 (Figure 3C), providing a simple explanation for the population of synapses with an OSI centered around 0.31. The shape of receptive field centers could not, however, explain the second population of synapses with OSIs approaching 1 (Figure 2C).

The second potential mechanism for generating pattern-detecting synapses invokes lateral inhibition from amacrine cells (Figure 3A), which contact bipolar cell terminals through GABAergic connections (Masland, 2012). The first evidence to support this model came when we measured OSIs using gratings of different spatial frequency and found that the receptive field centers of bipolar cell synapses were 4-fold smaller than the spatial patterns that they signaled most strongly (Figure 3D; Figure S4). The obvious candidates for neurons within the inner retina with large receptive fields are wide-field amacrine cells, so we tested their role by injecting a mixture of gabazine and strychnine into the intravitreal chamber to antagonize GABAergic and glycinergic receptors. With inhibition blocked, only 10% of 1,173 terminals displayed orientation selectivity, compared to 24.4% of 1,053 terminals with inhibition intact (Figure 3F; Figure S5). Notably, the population of bipolar cell terminals with OSIs around 1 was almost completely abolished (Figure 3E), indicating that they were indeed generated by lateral inhibition. Blocking inhibition also flattened the distribution of preferred orientations from one in which terminals tuned to the vertical were strongly overrepresented to one in which horizontal orientations were represented at slightly higher frequencies (Figure 3F).

Further evidence for the idea that inhibitory inputs contribute to the tuning of bipolar cell synapses is shown in Figure 4, which plots SyGCaMP6f responses in terminals displaying differing degrees of selectivity for the vertical and horizontal. In synapses with an OSI of 1, a grating orthogonal to the preferred orientation often caused a significant *decrease* in calcium below the baseline, indicating that a stimulus at the non-preferred orientation activated a counteracting inhibitory signal. These antagonistic responses depended on the spatial frequency of the stimulus (Figure S5) and were observed in 29% of terminals using a grating of 0.03 cycles per degree but only 9% of terminals using 0.121 cycles per degree, again indicating the involvement of amacrine cells with larger dendritic fields. When inhibition was blocked using gabazine and strychnine, responses with decreases in calcium for the null direction were completely abolished (data not shown), and the distribution of preferred orientations was flattened (Figure 3F).

Direct evidence for the idea that amacrine cells can also be orientation-selective was obtained by measuring their synaptic

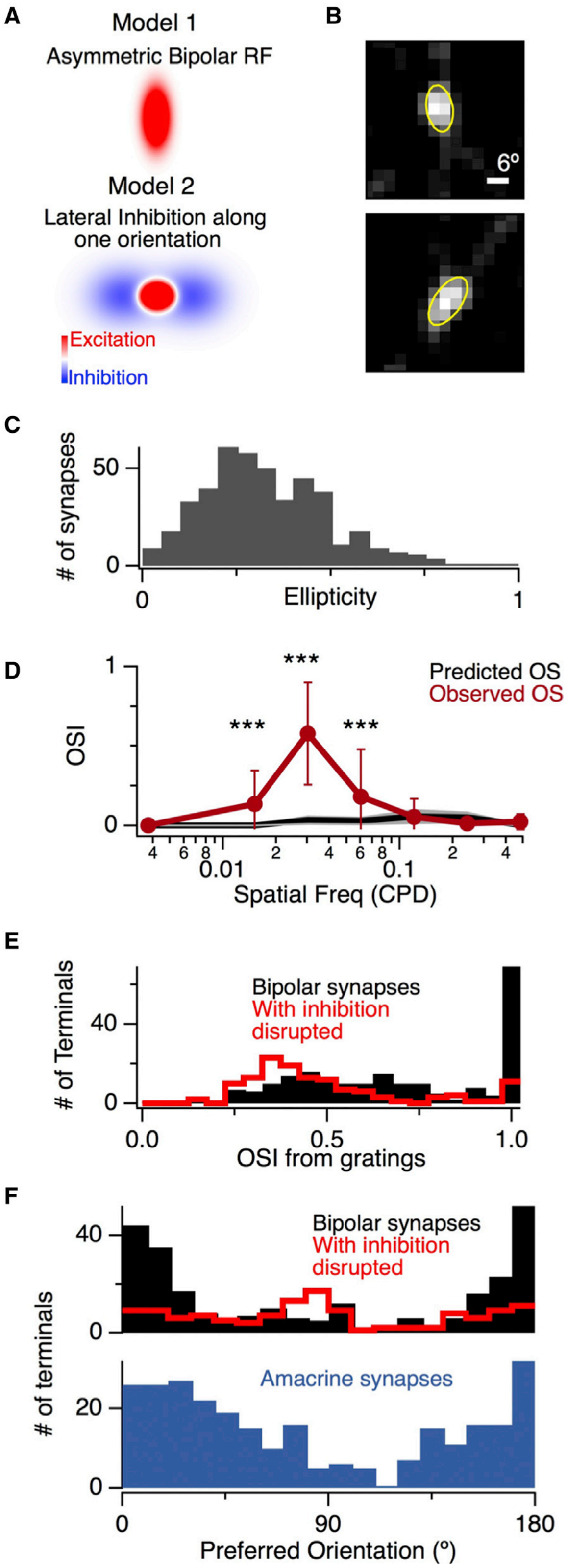

Figure 3. Intrinsic and Extrinsic Factors Generating Orientation Selectivity in Bipolar Cell Synapses

(A) Two mechanisms potentially generating orientation selectivity. In model 1, the synapse has an elongated receptive field center (red), which would lead to an orientation preference (for vertical stimuli in this example). Model 2 shows a synap receptive field (red) receiving inhibitory inputs that are stronger along the horizontal axis, which would also lead to vertical selectivity in the bipolar cell synapse.

(B) Examples of receptive field centers mapped in synapses of two separate bipolar cells. Yellow lines show a fit with a 2D Gaussian used to estimate the lengths of their major and minor axes.

(C) Histogram of the receptive field ellipticity measured from 442 bipolar cell synapses.

(D) The OSI as a function of spatial frequency in bipolar cell synapses (red, n = 819) compared with the OSI predicted from the receptive field dimensions assuming linearity (black, n = 442), displayed as mean ± SD.

(E) Comparison of the distributions of OSI under normal conditions (black, n = 257) and that remaining after disrupting inhibition with intravitreal injection of gabazine and strychnine (red, n = 127).

(F) Top: distribution of preferred orientations under normal conditions (black) and with inhibition disrupted (red). Bottom: distribution of preferred orientations of 274 amacrine cell synapses showing significant OS (orientation selective). On average, amacrine cells were activated more strongly by the vertical (0°) compared to the horizontal.

activity using SyGCaMP3.5 under the *ptf1a* promoter (Nikolaev et al., 2013). Across a population of 982 synapses, 274 displayed a significant degree of orientation selectivity with an overrepresentation of tuning to the vertical (Figure 3F, bottom). Amacrine cells are excited solely through bipolar cells, so one possibility is that they inherit their orientation selectivity from these inputs. If this mechanism were common across the population of amacrine cells, then they would be expected to exhibit an average distribution of preferred orientations similar to bipolar cells, and this was the observation (compare top and bottom in Figure 3F). The distribution of preferred orientations in amacrine cells was, however, significantly broader than in bipolar cell synapses, likely reflecting the contribution of other factors, such as intrinsic asymmetries in amacrine cell dendritic trees (Antinucci et al., 2016). Together, the results in Figures 3 and 4 demonstrate that lateral antagonism in the orientation domain helps generate bipolar cell synapses with the highest degree of orientation selectivity.

The Wiring of Spatial Pattern Detectors to Retinal Ganglion Cells

A key element of the pattern detector hypothesis is that individual RGCs receive inputs with a variety of tunings (Gollisch and Meister, 2010; Hosoya et al., 2005; Lesica et al., 2007). To investigate whether such RGCs exist in the retina of zebrafish, we used iGluSnFR to make a functional assessment of the rules governing bipolar cell-to-RGC connectivity. We sampled 27 RGCs, measuring from an average of 22 synaptic inputs in each, distributed over several focal planes. For each RGC, we tested whether the pattern of inputs of different types might reflect random sampling. First, each bipolar cell synapse in our complete sample of 1,053 recorded with SyGCaMP6f (Figure S3) was classified as being either tuned or untuned to orientation (STAR Methods). Next, the observed distribution of inputs to an RGC from which N synapses were imaged was compared with the distribution predicted by chance (estimated by

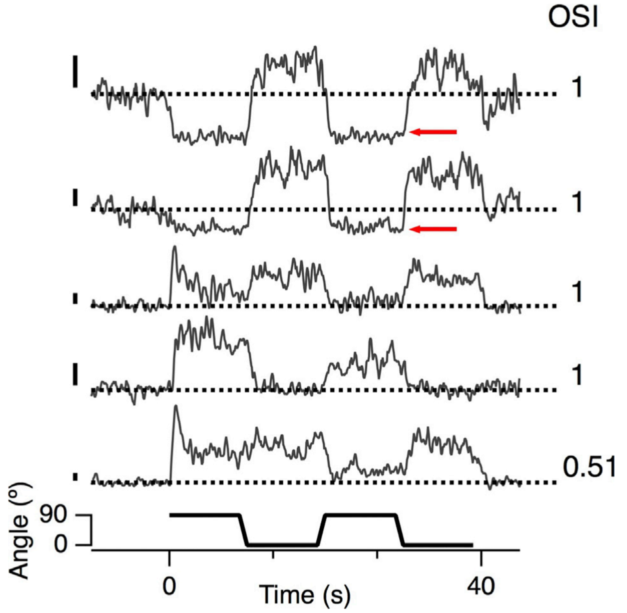

Figure 4. Lateral Antagonism in the Orientation Domain, Acting on Bipolar Cell Synapses

Shown are examples of presynaptic calcium transients measured in bipolar cell synapses using SyGCaMP6f. The stimulus is the same as used in Figures 1 and 2. The OSI for the top four terminals was 1. The upper two exhibited a fall in calcium, reflecting hyperpolarization (red arrows), when the stimulus switched to the non-preferred orientation, indicating increased inhibition. Note that this measurement cannot distinguish increased inhibition from decreased excitation unless the inhibition is sufficient to decrease presynaptic calcium below basal levels. All scale bars show 50% ΔF/F.

taking N synapses from the 1,053 at random and repeating 100,000 times). Twenty-three of the 27 RGCs had distributions of tuned and untuned inputs occurring with a probability of less than 1% compared with chance, indicating specific rather than random connectivity.

To explore connectivity patterns in more detail, we calculated for each RGC the ratio of inputs with a vertical preference to those with a horizontal preference ($R_{0/90}$), and the distribution is shown in Figure 5A. Two general patterns were observed. RGCs either received inputs weighted toward one orientation (the two extremes in the distribution; n = 19), or they received a mixture of excitation tuned to a variety of different orientations or none (the middle group in the distribution; n = 8). RGCs strongly selective for one of the two cardinal orientations, such as the example shown in Figure 5B, are likely to account for the population that provides outputs to the tectum that are statically tuned (Figure 1E, red). Two RGCs did not show any vertical-to-horizontal preference because none of the inputs were tuned, as shown by the example in Figure 5C, but the remaining 6 RGCs with $R_{0/90}$ around 0.5 received inputs of mixed tuning. Two examples of this last group of RGCs are shown in Figures 5D and 5E; these display the basic wiring proposed by the pattern detector hypothesis—convergence of input signals encoding different orientations. Averaging across all synapses sampled on each cell (gray traces in Figures 5D and 5E) again demonstrated that these driving signals were slow to adapt and could not, therefore, account for the rapid suppression of the output measured in the tectum.

A High-Pass Filter in Retinal Ganglion Cells

To investigate further the transformation of the visual signal within individual RGCs, we used iGluSnFR to compare inputs at their dendrites with the output transmitted from their axons. The similarity between the two signals was first quantified as a correlation (R^2) value, as would be used to assess a linear regression model. In 3 cells, the excitatory input alone was a very good predictor of axon output, accounting for ~66% of the variance, and two examples are shown in Figure 6A. It appears that inhibitory signals received by these RGCs, either within the retina or tectum, play a relatively minor role in shaping the final output. In the remaining five RGCs, R^2 averaged just 0.102 (Figure 6B), and the output resembled a high-pass-filtered version of the excitatory input; two examples are shown in Figures 6D and 6F. These RGCs signaled changes in orientation strongly but attenuated the sustained component as well as the 5 Hz flicker, as shown by the spectrograms in Figures 6E and 6G. A second indicator that a high-pass filter operation occurred in RGCs was observed when comparing the time to peak of the input and output signals elicited by a change in orientation; the output reached a peak 200 ms before the input (Figure 6C). Such a phase lead is a basic characteristic of a high-pass filter (Saggio, 2014).

Might the high-pass filter operating in some RGCs reflect their connectivity to the other key element of the inner retina, the amacrine cells? A general feature of the retinal connectome is local feedforward inhibition (FFI), in which a bipolar cell excites both an RGC and an amacrine cell that inhibits the same RGC, often on the same dendrite (Chen et al., 2010; Diamond, 2017; Johnston and Lagnado, 2015; Figure 7A). FFI is also a fundamental feature of the hippocampus and neocortex, where it controls the temporal window for firing in pyramidal neurons (Isaacson and Scanziani, 2011; Pouille et al., 2009) and can generate a high-pass filter (Milstein et al., 2015). Unfortunately, testing the role of FFI by pharmacological manipulation was confounded by the simultaneous block of lateral inhibition, which caused large increases in the gain of excitation through bipolar cells. We therefore used a combination of electrophysiology and modeling to assess the potential role of FFI. In model 1 (red), FFI was introduced as a high-pass filter, and rectification in the signal transmitted from bipolar cells to output of RGCs was introduced as a static threshold followed by a linear function. It is also possible that processes within the RGC, such as spike generation, introduce a threshold followed by a supra-linear scaling (Schwartz and Rieke, 2011; Turner and Rieke, 2016). We therefore also investigated model 2, in which the static non-linearity comprised a threshold followed by a power function (blue in Figure 7A, where the power is two).

The high-pass filter in model 1 was estimated using a multi-compartment model of a simplified RGC into which we integrated the properties of excitation and inhibition measured electrophysiologically in RGCs from goldfish (Johnston and Lagnado, 2015). Figure 7Bi shows how excitation in the model RGC became briefer as the strength of the inhibitory input was increased. The ratio of inhibitory to excitatory synapses varies between RGCs, so we used the mean ratio of 2:1 measured

experimentally (Johnston and Lagnado, 2015; Figure 7Bii). A key assumption of this model was that the excitatory current injected into the RGC dendrite is directly proportional to the iGluSnFR signal, which has been confirmed experimentally (Marvin et al., 2013). The only parameter that was not empirically determined was the threshold for the non-linearity. In tests of the model, we set this threshold to be 3 times the noise in the baseline.

Model 1, based on a high-pass filter, succeeded in two aspects where model 2 failed. First, it predicted that the output from an RGC has an earlier time to peak than the synaptic input, as observed experimentally (Figures 6C, 6D, and 6F). In contrast, model 2, based on a power function, predicted a peak output that was delayed. When this power was two, the median delay was 0.23 s relative to the actual measurement (Figures 7A and 7E), and adjustments of both the threshold and exponent of the nonlinearity failed to account for the phase lead in RGC output. Second, and more qualitatively, the FFI model highlighted transients in the input more clearly. For instance, Figure 7C shows an example RGC (statically tuned) in which the glutamatergic input fluctuates rapidly (black trace); these fluctuations are apparent in the measured output (gray) and are also predicted by the FFI model (red) but not by a squaring non-linearity (blue).

An example of the output of the models for an RGC receiving a mixture of inputs tuned to different orientations is shown in Figure 7D; model 1 predicted a clear output signal at each change in orientation, as observed in RGCs displaying a predictive code, but model 2 did not. Furthermore, although the decay time constant of the initial response in model 2 could be shortened to match the RGC output by tuning the threshold and exponent of the nonlinearity, this was always at the expense of reducing or eliminating transients evoked by subsequent orientation changes, as is evident in Figures 7A and 7D. We conclude that a high-pass filter, most likely generated by FFI, is a key mechanism by which maintained excitation received by RGCs is nulled to prevent the transmission of unchanging signals to the tectum.

DISCUSSION

A basic constraint in the design of neural circuits is the need to transmit information in an energy-efficient manner, and removing redundancies from incoming signals is one of the most important ways to achieve this (Barlow, 1961; Niven and Laughlin, 2008; Srinivasan et al., 1982; Sterling and Laughlin, 2015). In this study we have delineated a circuit that allows individual neurons to signal

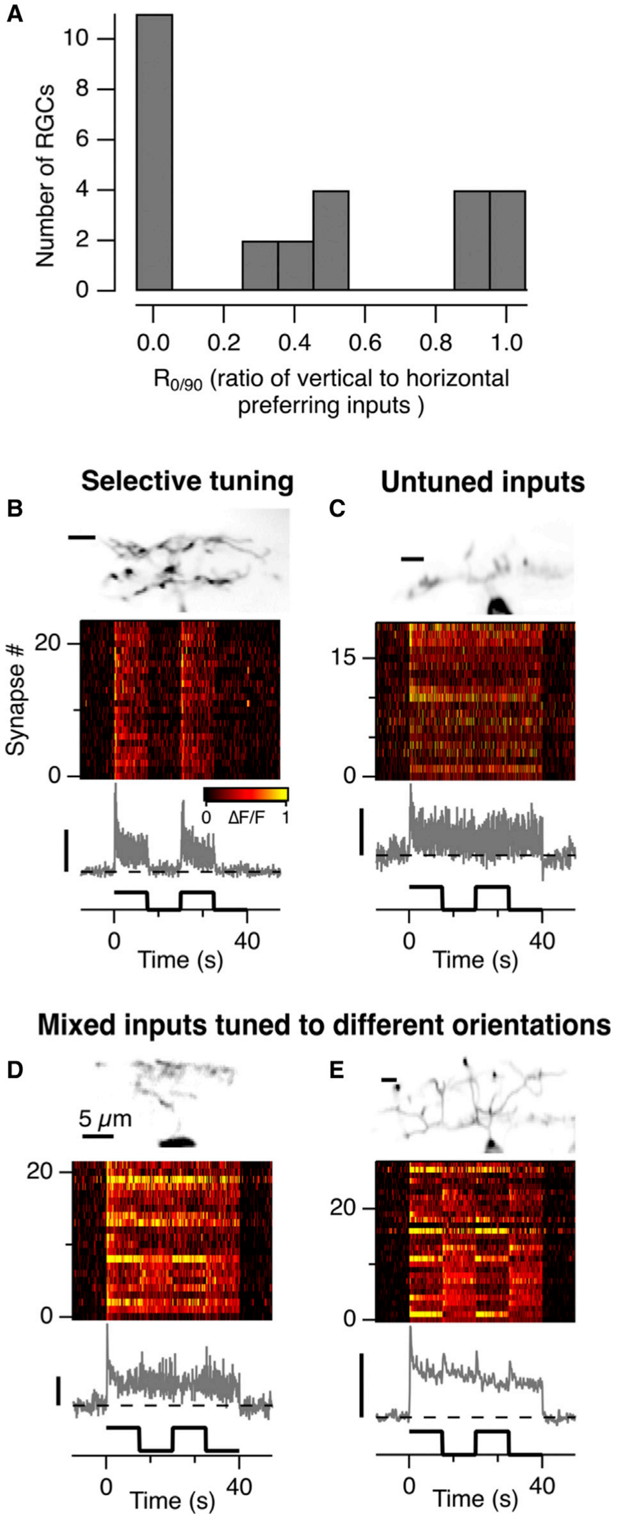

Figure 5. The Wiring of Bipolar Cell Synapses and Retinal Ganglion Cells

(A) Distribution of values of $R_{0/90}$, the ratio of inputs with a vertical preference to those with a horizontal preference (n = 27 RGCs).

(B) An example of the synaptic inputs to one of 19 retinal RGCs that were selectively wired to synapses tuned to a similar orientation. The gray trace shows the average of these 24 inputs; the OSI averaged 0.98.

(C) An example of the synaptic inputs to one of 2 RGCs in which none of the inputs were orientation-selective. The gray trace shows the average of these 19 inputs.

(D) An example of an RGC receiving a mix of excitatory inputs tuned to 0° (8), 90° (4), and non-OS (10).

(E) Another example of an RGC receiving a mix of excitatory inputs tuned to 0° (13), 90° (7), and non-OS (9). Note that the average excitatory drive to these cells shows a transient increase at each change in orientation.

All image scale bars, 5 μm; all ΔF/F scale bars, 50%.

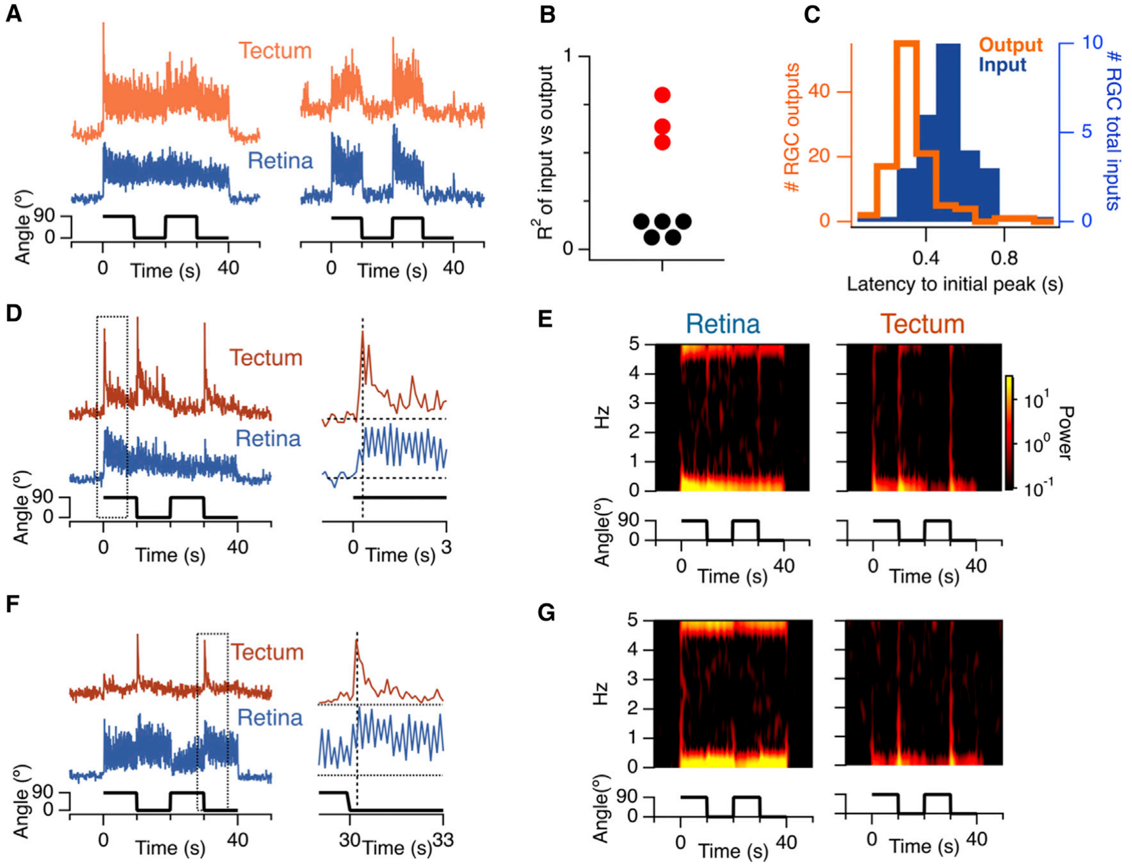

Figure 6. A High-Pass Filter Operates in Some RGCs

(A) Examples of the averaged excitatory input (blue) and glutamatergic output (orange) of two RGCs in which input and output were strongly correlated (stimulus as in Figures 1 and 2). The RGC on the right was statically tuned (OSI = 1).

(B) R^2 values for the correlation between the total excitatory drive and output for 8 different RGCs. The cells in (A) are marked in red, and output closely resembled input. The cells marked in black (which includes those in D–F) are those in which the output was markedly different from the input.

(C) Comparison of the latency between a switch in the orientation of the stimulus and the first peak in the iGluSnFR signal on the dendrites (input, blue) and at the axon (output, orange). On average, the output leads the input by ~200 ms, indicative of a high-pass filter.

(D and F) Two examples of RGCs showing low degrees of correlation (R^2) between excitatory input and output. The dashed box for each cell is enlarged on the right, illustrating the filtering out of the sustained component of the input. The inputs were more strongly orientation-selective in (F) compared to (D).

(E and G) Spectrogram showing the power in the signals in (D) and (F) as a function of frequency (ordinate) and time (abscissa). Note that that the outputs in the tectum contain less power over a range of frequencies up to 5 Hz.

changes in spatial patterns while strongly suppressing the transmission of unchanging and, therefore, redundant information. This implementation of a dynamic predictive code involves re-tuning of orientation-selective RGCs, as proposed by Hosoya et al. (2005), by the circuit shown in Figure 8. The basic elements of this circuit are: (1) bipolar cell synapses acting as pattern detectors because of their intrinsic orientation selectivity (Figure 2; Figure S3) and because of inhibitory inputs that act presynaptically to generate lateral antagonism in the orientation domain (Figures 3 and 4); (2) a proportion of amacrine cells reflecting the orientation tuning of the bipolar cells that drive them (Figure 3F); (3) individual RGCs receiving a mixture of excitatory inputs tuned to different orientations (Figure 5D), and (4) FFI through amacrine cells onto RGCs to generate a high-pass filter (Figures 6 and 7).

The basic features of the circuit shown in Figure 8 are recapitulated in other parts of the brain that are thought to carry out some form of dynamic predictive coding, such as the primary visual and auditory cortices (Bock et al., 2011; Priebe, 2016; Smith and Lewicki, 2006), the hippocampus (Mori et al., 2007), and the optic tectum (Luksch et al., 2004), and it may therefore reflect a general strategy by which excitatory and inhibitory neurons are connected to highlight transient inputs while nulling steady signals. For instance, individual pyramidal cells in the primary visual cortex (V1) also receive excitatory synaptic inputs tuned to a variety of different orientations (Iacaruso et al., 2017), and their tuning shifts dynamically as they adapt to the distribution of orientations within a recent period of stimulation (Benucci et al., 2013). It is less clear whether changes in orientation tuning within V1 reflect primarily depression within excitatory synapses (Müller et al., 1999) or the additional effects of inhibition (Priebe, 2016). In the retina, synaptic depression and FFI both reduce the net excitation caused by an input of a particular orientation but

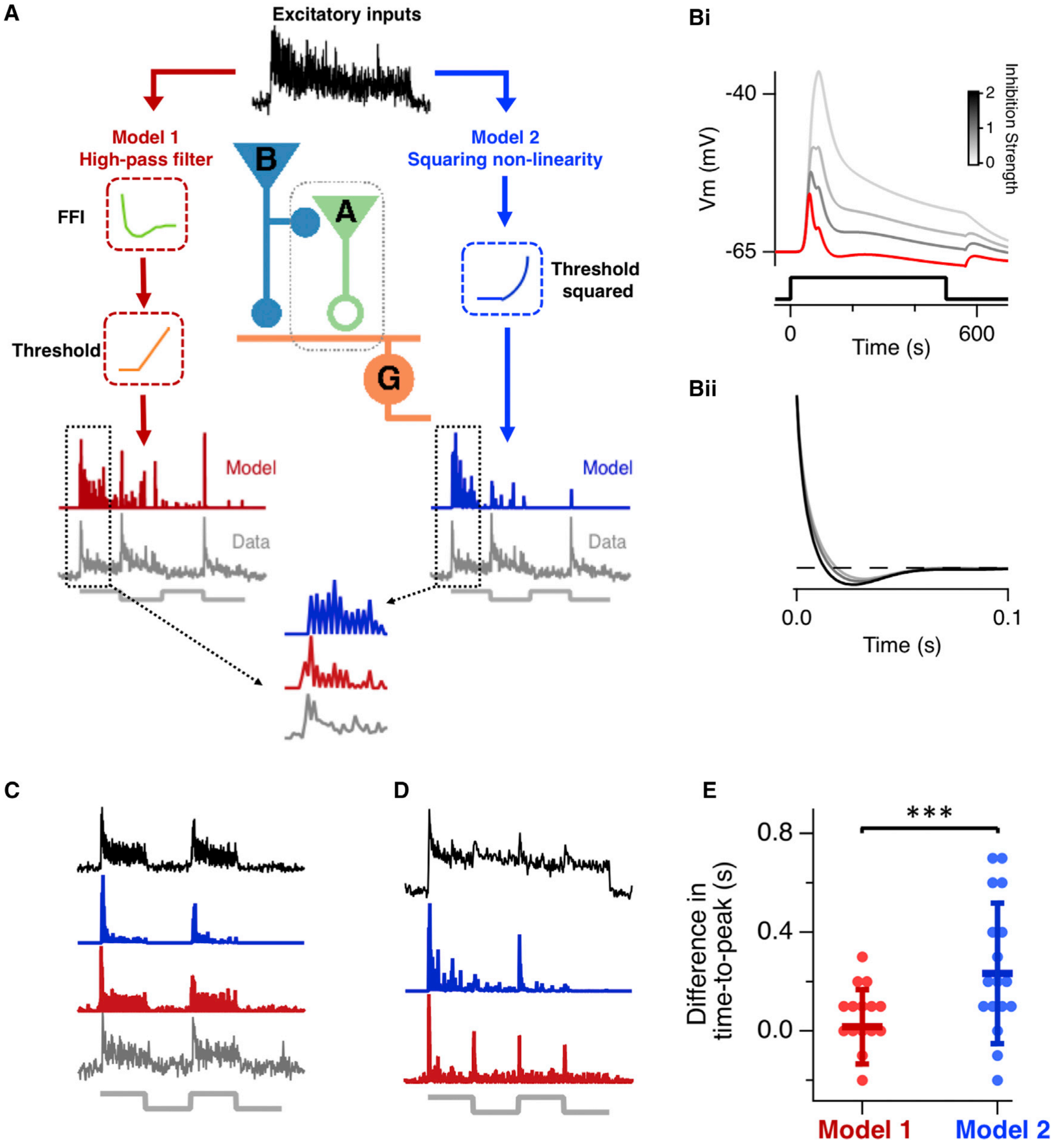

Figure 7. Feedforward Inhibition Can Account for the Transient Outputs of RGCs Generating a Dynamic Predictive Code

(A) Comparison of two models. In model 1, an RGC (G) receives excitatory inputs from bipolar cells (B), which also activate local FFI through amacrine cells (A) contacting the same dendrite. The input to the model is the average excitatory input measured using iGluSnFR (black trace), which is convolved with the high-pass filter calculated in (Bii) (green) and then passed through a static non-linearity (orange). The output of the model (red) can be compared with the output of the same RGC, measured using iGluSnFR (gray). In model 2, there is no high-pass filter, and the input instead passes through a squaring static non-linearity (blue). In this example, the non-linearity is a threshold followed by squaring. Note that the transients in the measured output are reproduced by model 1 but not model 2. The initial parts of the traces enclosed by the dashed boxes are enlarged below; the time-to-peak in the output is predicted by model 1 but not model 2.

(Bi) The membrane potential of an RGC in response to step activation of the excitatory and inhibitory synapses modeled in NEURON. Increasing the strength of inhibition makes the response more transient.

(Bii) The impulse response of the temporal filter caused by FFI shown by the red trace in (B). This filter assumed a 2:1 ratio of inhibitory to excitatory synapses.

(C) Examples of the output of model 1 (red) and model 2 (blue) for an RGC receiving inputs tuned to a similar orientation (average synaptic iGluSnFR signal are shown in black).

(D) An example of the output of the models for an RGC receiving a mixture of inputs tuned to different orientations. Model 1 predicted a clear signal at each change in orientation but model 2 did not.

(E) The difference between the measured and predicted time to peak of the transient, measured after a change in orientation. Each point represents a different RGC. The median difference of model 1 was zero, whereas model 2, incorporating a power of two, was significantly delayed, with a median of 0.23 ± 0.28 s (Wilcoxon signed-rank test, $p = 0.002$). Error bars show median absolute difference.

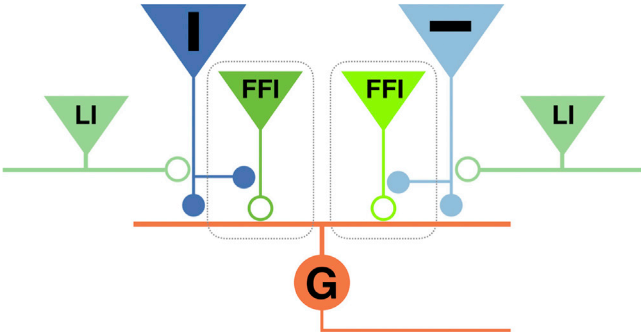

Figure 8. A Retinal Circuit Generating a Dynamic Predictive Code for Orientated Features

The RGC (G) receives a mixture of bipolar cell inputs tuned to different orientations, including those preferentially signaling the vertical (dark blue) and horizontal (light blue). Bipolar cell synapses tuned to orientation receive lateral inhibition (LI) from amacrine cells (green) along their orthogonal orientation. Amacrine cells provide FFI onto RGC dendrites, generating a high-pass filter that nulls unchanging signals. Excitatory synapses are represented by solid circles and inhibitory synapses by open circles.

with very different timescales; presynaptic depression usually decreases the gain of an input over timescales of several seconds (Figure 2D; Nikolaev et al., 2013), whereas FFI acts in a fraction of a second (Milstein et al., 2015). Another key variable is the strength of the FFI received by the neuron acting as the pattern detector. We found a distinct subset of RGCs that did not act as high-pass filters (Figure 6B), consistent with the idea that the FFI they receive is weak or non-existent. With variations, therefore, the basic circuit shown in Figure 8 could act with varying efficiency and on different timescales to alter tuning and suppress transmission of maintained signals.

We found that most RGCs in zebrafish do not completely suppress an unchanging input and, therefore, operate with an appreciable degree of redundancy. Similarly, a survey of the retinas of salamanders and rabbits found that only half of the RGCs adapt to an orientated stimulus, with gain changes averaging a factor of about two (Hosoya et al., 2005). The lack of more complete adaptation can now be understood in terms of the excitatory inputs RGCs receive from bipolar cells, which depress incompletely and relatively slowly while a stimulus is maintained (Figure 2). Modeling basic features of connectivity in the inner retina indicates that the steady excitatory input is only nulled effectively and rapidly in the subset of RGCs that additionally receive FFI (Figures 6 and 7). The tectum, therefore, receives information about spatial patterns in at least two ways; some RGCs signal changes toward a preferred orientation and are stably tuned, whereas others signal changes in any orientation and retune rapidly (Figure 1E).

How are these different signaling modes used by downstream circuits? Answering such questions will be aided by measuring the distribution of spatial correlations in the orientation space a zebrafish is encountering in its normal environment of shallow, slow-moving streams. Among the first pattern detectors, the bipolar cell synapses, there is an overrepresentation of vertical orientations (Figure 2F) that might, for instance, reflect the importance of detecting vegetation growing upward toward sunlight.

It is also possible that RGCs generating a dynamic predictive code for orientation serve to alert the animal to rotations of its body axis relative to the surrounds, in particular relative to the preferred (vertical) orientation. It is known that the righting reflex is driven by inputs from the visual system as well as the vestibular and somatosensory systems (Kalueff et al., 2013), and signaling sudden changes in spatial patterns may serve to detect changes in body roll.

The integration of anatomical and physiological studies of neural circuits has been termed "functional connectomics" (Seung, 2011), and this idea has generally been framed in terms of defining the rules that relate the functional properties of neurons with the synaptic connections they make. The present study highlights the importance complementing this information with an understanding of the signals that are actually transmitted within circuits. The use of fluorescent reporters of synaptic activity, such as iGluSnFR, provides a promising approach for understanding one of the most basic aspects of any neural circuit: the input-output relations of the neurons within it.

STAR★METHODS

Detailed methods are provided in the online version of this paper and include the following:

- KEY RESOURCES TABLE
- CONTACT FOR REAGENT AND RESOURCE SHARING
- EXPERIMENTAL MODEL AND SUBJECT DETAILS
  - Zebrafish
- METHOD DETAILS
  - Molecular Biology
  - Visual stimuli
  - Two-photon imaging
  - Drug application
- QUANTIFICATION AND STATISTICAL ANALYSIS
  - Image segmentation
  - Classification of retinal ganglion cells
  - Quantifying orientation selectivity
  - Analysis of responses to moving bars
  - Receptive field reconstruction
  - Modeling the contribution of RF ellipticity to OSI
  - Modeling the filter resulting from feedforward inhibition

SUPPLEMENTAL INFORMATION

Supplemental Information can be found online at https://doi.org/10.1016/j.neuron.2019.04.002.

ACKNOWLEDGMENTS

We thank H. Smulders and N. Bashford for looking after our zebrafish and Herwig Baier and Henry Roehl for gifting plasmids. This work was supported by a grant from the Wellcome Trust (102905/Z/13/Z to L.L.).

AUTHOR CONTRIBUTIONS

J.J. conceived the project, designed and carried out experiments, analyzed results, and prepared the manuscript. S.-H.S. carried out molecular biology and transgenesis, performed experiments, analyzed results, and prepared the manuscript. L.S.A.D. performed experiments. S.R. and M.O. provided the

UAS:iGluSnFR line and commented on the manuscript. L.L. conceived the project, designed experiments, analyzed results, and prepared the manuscript.

DECLARATION OF INTERESTS

The authors declare no competing interests

Received: June 19, 2018
Revised: February 15, 2019
Accepted: March 28, 2019
Published: May 1, 2019

REFERENCES

Antinucci, P., Suleyman, O., Monfries, C., and Hindges, R. (2016). Neural mechanisms generating orientation selectivity in the retina. Curr. Biol. *26*, 1802–1815.

Asari, H., and Meister, M. (2012). Divergence of visual channels in the inner retina. Nat. Neurosci. *15*, 1581–1589.

Atick, J.J., and Redlich, A.N. (1992). What does the retina know about natural scenes? Neural Comput. *4*, 196–210.

Barlow, H.B. (1961). Possible principles underlying the transformations of sensory messages. In Sensory Communication, W.A. Rosenblith, ed. (MIT Press), pp. 217–234.

Bastos, A.M., Usrey, W.M., Adams, R.A., Mangun, G.R., Fries, P., and Friston, K.J. (2012). Canonical microcircuits for predictive coding. Neuron *76*, 695–711.

Ben Fredj, N., Hammond, S., Otsuna, H., Chien, C.B., Burrone, J., and Meyer, M.P. (2010). Synaptic activity and activity-dependent competition regulates axon arbor maturation, growth arrest, and territory in the retinotectal projection. J. Neurosci. *30*, 10939–10951.

Benucci, A., Saleem, A.B., and Carandini, M. (2013). Adaptation maintains population homeostasis in primary visual cortex. Nat. Neurosci. *16*, 724–729.

Bock, D.D., Lee, W.C., Kerlin, A.M., Andermann, M.L., Hood, G., Wetzel, A.W., Yurgenson, S., Soucy, E.R., Kim, H.S., and Reid, R.C. (2011). Network anatomy and in vivo physiology of visual cortical neurons. Nature *471*, 177–182.

Brainard, D.H. (1997). The Psychophysics Toolbox. Spat. Vis. *10*, 433–436.

Branco, T., and Häusser, M. (2011). Synaptic integration gradients in single cortical pyramidal cell dendrites. Neuron *69*, 885–892.

Chen, X., Hsueh, H.A., Greenberg, K., and Werblin, F.S. (2010). Three forms of spatial temporal feedforward inhibition are common to different ganglion cell types in rabbit retina. J. Neurophysiol. *103*, 2618–2632.

Diamond, J.S. (2017). Inhibitory interneurons in the retina: types, circuitry, and function. Annu. Rev. Vis. Sci. *3*, 1–24.

Distel, M., Wullimann, M.F., and Köster, R.W. (2009). Optimized Gal4 genetics for permanent gene expression mapping in zebrafish. Proc. Natl. Acad. Sci. USA *106*, 13365–13370.

Dragoi, V., Sharma, J., and Sur, M. (2000). Adaptation-induced plasticity of orientation tuning in adult visual cortex. Neuron *28*, 287–298.

Ekman, M., Kok, P., and de Lange, F.P. (2017). Time-compressed preplay of anticipated events in human primary visual cortex. Nat. Commun. *8*, 15276.

Fairhall, A.L., Lewen, G.D., Bialek, W., and de Ruyter Van Steveninck, R.R. (2001). Efficiency and ambiguity in an adaptive neural code. Nature *412*, 787–792.

Gollisch, T., and Meister, M. (2010). Eye smarter than scientists believed: neural computations in circuits of the retina. Neuron *65*, 150–164.

Graham, N.V.S. (2001). Visual Pattern Analyzers (Oxford University Press).

Hindy, N.C., Ng, F.Y., and Turk-Browne, N.B. (2016). Linking pattern completion in the hippocampus to predictive coding in visual cortex. Nat. Neurosci. *19*, 665–667.

Hosoya, T., Baccus, S.A., and Meister, M. (2005). Dynamic predictive coding by the retina. Nature *436*, 71–77.

Hubel, D.H., and Wiesel, T.N. (1959). Receptive fields of single neurones in the cat's striate cortex. J. Physiol. *148*, 574–591.

Iacaruso, M.F., Gasler, I.T., and Hofer, S.B. (2017). Synaptic organization of visual space in primary visual cortex. Nature *547*, 449–452.

Isaacson, J.S., and Scanziani, M. (2011). How inhibition shapes cortical activity. Neuron *72*, 231–243.

Jarsky, T., Cembrowski, M., Logan, S.M., Kath, W.L., Riecke, H., Demb, J.B., and Singer, J.H. (2011). A synaptic mechanism for retinal adaptation to luminance and contrast. J. Neurosci. *31*, 11003–11015.

Johnston, J., and Lagnado, L. (2015). General features of the retinal connectome determine the computation of motion anticipation. eLife *4*, e06250.

Johnston, J., Ding, H., Seibel, S.H., Esposti, F., and Lagnado, L. (2014). Rapid mapping of visual receptive fields by filtered back projection: application to multi-neuronal electrophysiology and imaging. J. Physiol. *592*, 4839–4854.

Kalueff, A.V., Gebhardt, M., Stewart, A.M., Cachat, J.M., Brimmer, M., Chawla, J.S., Craddock, C., Kyzar, E.J., Roth, A., Landsman, S., et al.; Zebrafish Neuroscience Research Consortium (2013). Towards a comprehensive catalog of zebrafish behavior 1.0 and beyond. Zebrafish *10*, 70–86.

Kastner, D.B., and Baccus, S.A. (2011). Coordinated dynamic encoding in the retina using opposing forms of plasticity. Nat. Neurosci. *14*, 1317–1322.

Kastner, D.B., and Baccus, S.A. (2014). Insights from the retina into the diverse and general computations of adaptation, detection, and prediction. Curr. Opin. Neurobiol. *25*, 63–69.

Kawakami, K. (2007). Tol2: a versatile gene transfer vector in vertebrates. Genome Biol. *8* (*Suppl 1*), S7.

Kingston, A., and Autrusseau, F. (2008). Lossless image compression via predictive coding of discrete Radon projections. Signal Process. Image Commun. *23*, 313–324.

Kondo, S., and Ohki, K. (2016). Laminar differences in the orientation selectivity of geniculate afferents in mouse primary visual cortex. Nat. Neurosci. *19*, 316–319.

Lesica, N.A., Jin, J., Weng, C., Yeh, C.I., Butts, D.A., Stanley, G.B., and Alonso, J.M. (2007). Adaptation to stimulus contrast and correlations during natural visual stimulation. Neuron *55*, 479–491.

Li, Y.N., Tsujimura, T., Kawamura, S., and Dowling, J.E. (2012). Bipolar cell-photoreceptor connectivity in the zebrafish (Danio rerio) retina. J. Comp. Neurol. *520*, 3786–3802.

Luksch, H., Khanbabaie, R., and Wessel, R. (2004). Synaptic dynamics mediate sensitivity to motion independent of stimulus details. Nat. Neurosci. *7*, 380–388.

Marvin, J.S., Borghuis, B.G., Tian, L., Cichon, J., Harnett, M.T., Akerboom, J., Gordus, A., Renninger, S.L., Chen, T.W., Bargmann, C.I., et al. (2013). An optimized fluorescent probe for visualizing glutamate neurotransmission. Nat. Methods *10*, 162–170.

Masland, R.H. (2012). The neuronal organization of the retina. Neuron *76*, 266–280.

Milstein, A.D., Bloss, E.B., Apostolides, P.F., Vaidya, S.P., Dilly, G.A., Zemelman, B.V., and Magee, J.C. (2015). Inhibitory gating of input comparison in the CA1 microcircuit. Neuron *87*, 1274–1289.

Mori, M., Gähwiler, B.H., and Gerber, U. (2007). Recruitment of an inhibitory hippocampal network after bursting in a single granule cell. Proc. Natl. Acad. Sci. USA *104*, 7640–7645.

Müller, J.R., Metha, A.B., Krauskopf, J., and Lennie, P. (1999). Rapid adaptation in visual cortex to the structure of images. Science *285*, 1405–1408.

Nath, A., and Schwartz, G.W. (2017). Electrical synapses convey orientation selectivity in the mouse retina. Nat. Commun. *8*, 2025.

Nikolaev, A., Leung, K.M., Odermatt, B., and Lagnado, L. (2013). Synaptic mechanisms of adaptation and sensitization in the retina. Nat. Neurosci. *16*, 934–941.

Nikolaou, N., Lowe, A.S., Walker, A.S., Abbas, F., Hunter, P.R., Thompson, I.D., and Meyer, M.P. (2012). Parametric functional maps of visual inputs to the tectum. Neuron *76*, 317–324.

Nirenberg, S., Bomash, I., Pillow, J.W., and Victor, J.D. (2010). Heterogeneous response dynamics in retinal ganglion cells: the interplay of predictive coding and adaptation. J. Neurophysiol. *103*, 3184–3194.

Niven, J.E., and Laughlin, S.B. (2008). Energy limitation as a selective pressure on the evolution of sensory systems. J. Exp. Biol. *211*, 1792–1804.

Odermatt, B., Nikolaev, A., and Lagnado, L. (2012). Encoding of luminance and contrast by linear and nonlinear synapses in the retina. Neuron *73*, 758–773.

Ohki, K., Chung, S., Ch'ng, Y.H., Kara, P., and Reid, R.C. (2005). Functional imaging with cellular resolution reveals precise micro-architecture in visual cortex. Nature *433*, 597–603.

Parker, N.F., Cameron, C.M., Taliaferro, J.P., Lee, J., Choi, J.Y., Davidson, T.J., Daw, N.D., and Witten, I.B. (2016). Reward and choice encoding in terminals of midbrain dopamine neurons depends on striatal target. Nat. Neurosci. *19*, 845–854.

Peyrache, A., Lacroix, M.M., Petersen, P.C., and Buzsáki, G. (2015). Internally organized mechanisms of the head direction sense. Nat. Neurosci. *18*, 569–575.

Pologruto, T.A., Sabatini, B.L., and Svoboda, K. (2003). ScanImage: flexible software for operating laser scanning microscopes. Biomed. Eng. Online *2*, 13.

Portugues, R., Feierstein, C.E., Engert, F., and Orger, M.B. (2014). Whole-brain activity maps reveal stereotyped, distributed networks for visuomotor behavior. Neuron *81*, 1328–1343.

Pouille, F., Marin-Burgin, A., Adesnik, H., Atallah, B.V., and Scanziani, M. (2009). Input normalization by global feedforward inhibition expands cortical dynamic range. Nat. Neurosci. *12*, 1577–1585.

Priebe, N.J. (2016). Mechanisms of orientation selectivity in the primary visual cortex. Annu. Rev. Vis. Sci. *2*, 85–107.

Rao, R.P., and Ballard, D.H. (1999). Predictive coding in the visual cortex: a functional interpretation of some extra-classical receptive-field effects. Nat. Neurosci. *2*, 79–87.

Robles, E., Laurell, E., and Baier, H. (2014). The retinal projectome reveals brain-area-specific visual representations generated by ganglion cell diversity. Curr. Biol. *24*, 2085–2096.

Saggio, G. (2014). Principles of Analog Electronics (CRC Press).

Schmidt-Hieber, C., Toleikyte, G., Aitchison, L., Roth, A., Clark, B.A., Branco, T., and Häusser, M. (2017). Active dendritic integration as a mechanism for robust and precise grid cell firing. Nat. Neurosci. *20*, 1114–1121.

Schultz, W. (2016). Dopamine reward prediction-error signalling: a two-component response. Nat. Rev. Neurosci. *17*, 183–195.

Schwartz, G., and Rieke, F. (2011). Perspectives on: information and coding in mammalian sensory physiology: nonlinear spatial encoding by retinal ganglion cells: when $1 + 1 \neq 2$. J. Gen. Physiol. *138*, 283–290.

Seung, H.S. (2011). Neuroscience: towards functional connectomics. Nature *471*, 170–172.

Sharpee, T.O., Sugihara, H., Kurgansky, A.V., Rebrik, S.P., Stryker, M.P., and Miller, K.D. (2006). Adaptive filtering enhances information transmission in visual cortex. Nature *439*, 936–942.

Smith, E.C., and Lewicki, M.S. (2006). Efficient auditory coding. Nature *439*, 978–982.

Sohoglu, E., and Chait, M. (2016). Detecting and representing predictable structure during auditory scene analysis. eLife *5*, e19113.

Srinivasan, M.V., Laughlin, S.B., and Dubs, A. (1982). Predictive coding: a fresh view of inhibition in the retina. Proc. R. Soc. Lond. B Biol. Sci. *216*, 427–459.

Stachenfeld, K.L., Botvinick, M.M., and Gershman, S.J. (2017). The hippocampus as a predictive map. Nat. Neurosci. *20*, 1643–1653.

Sterling, P., and Laughlin, S.B. (2015). Principles of Neural Design (MIT Press).

Turner, M.H., and Rieke, F. (2016). Synaptic rectification controls nonlinear spatial integration of natural visual inputs. Neuron *90*, 1257–1271.

van Hateren, J.H. (1992). Real and optimal neural images in early vision. Nature *360*, 68–70.

Venkataramani, S., and Taylor, W.R. (2016). Synaptic mechanisms generating orientation selectivity in the ON pathway of the rabbit retina. J. Neurosci. *36*, 3336–3349.

Yonehara, K., Farrow, K., Ghanem, A., Hillier, D., Balint, K., Teixeira, M., Jüttner, J., Noda, M., Neve, R.L., Conzelmann, K.K., and Roska, B. (2013). The first stage of cardinal direction selectivity is localized to the dendrites of retinal ganglion cells. Neuron *79*, 1078–1085.

STAR★METHODS

KEY RESOURCES TABLE

REAGENT or RESOURCE	SOURCE	IDENTIFIER
Chemicals, Peptides, and Recombinant Proteins		
Gabazine	Tocris	Cat. No. 1262
Strychnine	Tocris	Cat. No. 2785
Alexa 594	ThermoFisher Scientific	A10438
Experimental Models: Organisms/Strains		
Zebrafish expressing SyGCaMP6 in retinal bipolar cells	Laboratory of Leon Lagnado	Tg(*-1.8ctbp2:SyGCaMP6*)
Zebrafish expressing SyGCaMP3 in retinal amacrine cells	Laboratory of Leon Lagnado	Tg(*ptf1a:gal4; UAS:SyGCaMP3*)
Zebrafish containing the UAS:iGluSnFR transgene	Laboratory of Michael Orger	Tg(*10xUAS:iGluSnFR*^{ccu003t})
Zebrafish homozygous for the roy;nacre double mutant	Laboratory of Leon Lagnado	*Casper*
Oligonucleotides		
Plasmid: 14xUAS:MCS in pT2KXIGin	Herwig Baier	N/A
Plasmid: P5E-HuC (elavl3)	Henry Roehl Lab	N/A
Plasmid: pCH-MCS-KalTA4GI	Herwig Baier	N/A
Plasmid: IsceI Ribeye:SyGCaMP6.10.500-Bleeding Heart	Leon Lagnado	N/A
Software and Algorithms		
Shapeshifter: Software for delivering visual stimuli	Jamie Johnston	https://github.com/JohnstonLab/Shapeshifter

CONTACT FOR REAGENT AND RESOURCE SHARING

Further information and requests for resources and reagents should be directed to and will be fulfilled by the Lead Contact, Leon Lagnado (L.Lagnado@sussex.ac.uk).

EXPERIMENTAL MODEL AND SUBJECT DETAILS

Zebrafish

All animal procedures were performed in accordance with UK Home Office guidelines and with the approval of the University of Sussex Animal Work Ethical Review Board.

Tg(*-1.8ctbp2:SyGCaMP6*) and Tg(*ptf1a:gal4; UAS:SyGCaMP3*) fish were generated as described previously (Odermatt et al., 2012). The Tg(*10xUAS:iGluSnFR*^{ccu003t}) was generated as follows. DNA coding for iGluSnFR (Marvin et al., 2013), in the Gateway destination vector pDestTol2pA (http://tol2kit.genetics.utah.edu/index.php/Main_Page; Kawakami, 2007), was cloned downstream of ten repeats of the Upstream Activation Sequence (10xUAS) using LR Gateway recombination. 12 ng/μl plasmid DNA and 40 ng/μl Tol2 transposase mRNA with 0.02% phenol red was injected into 1-cell stage embryos heterozygous for Isl2b:Gal4 (Ben Fredj et al., 2010). Transient and mosaic iGluSnFR-expression was achieved by injection of the HuCKalTA4 plasmid in combination with *Tol2* transposase (Kawakami, 2007) into 1-cell stage embryos derived from in crosses of Tg(*10xUAS:iGluSnFR*^{ccu003t}) which have been previously outcrossed to wild-type fish in order to split them from the Isl2b:Gal4 transgene. The concentration of the DNA and mRNA in the injection mix each were adjusted to 12.5 ng/ μl.

Fish were raised and maintained under a 14 h light/10 h dark cycle and standard conditions as described previously (Johnston et al., 2014). To aid imaging through the eye, fish used for 2-photon imaging were either heterozygous or homozygous for the *casper* mutations, which results in hypopigmentation due to the lack of melano- and iridophores. To further suppress pigmentation fish were treated with 1-phenyl-2-thiourea (200 μM final concentration; Sigma) from 1 day post fertilization to reduce pigmentation further. Fish were used at 6-8 days post fertilization.

METHOD DETAILS

Molecular Biology

The HuCKalTA4 plasmid was generated via the Gibson Assembly Cloning method. The plasmid features the HuC promoter that drives expression of the optimized transcriptional activator KalTA4 in most neurons in the brain, including amacrine cells and RGCs in the zebrafish retina. The optimization of Gal4 has been described previously (Distel et al., 2009). The plasmid contains a second small promotor (cmcl2) which drives expression of the mcherry fluorophore in the heart, this approach is commonly used in order to enable for phenotypic screening of transgenes that are not visible. Plasmid details are given in the Key Resources Table.

Visual stimuli

Visual stimuli were generated using Shapeshifter software (Johnston et al., 2014) written in MATLAB (MathWorks, Natick, MA, U.S.A.) utilizing Psychophysics Toolbox (Brainard, 1997). Stimuli were presented using an Optoma PK320 pico-projector (Optoma, Watford, Hertfordshire, UK), modified so that only the red LED was used for projection (Johnston et al., 2014). The mean irradiance of the screen was 12.7 nW mm^{-2} and fish were positioned so that they viewed the center of the screen and were adapted to the mean luminance for $\geq$ 10 mins. Square wave grating stimuli had an amplitude from -100% to +100% contrast and were designed so that the center of a bar was always aligned with the center of the screen, rather than an edge. For the spatial frequency tuning in Figure S4 and Figure 3D each frequency was presented separately and in a pseudorandom order, the resulting videos were then concatenated in order of spatial frequency. For the moving bar experiments visual stimuli consisted of -100% contrast bars with a width 4.1° of visual angle which traversed the screen at 18.6° s^{-1} in 8 different directions, thus giving stimuli of 8 directions and 4 orientations. The bar height spanned the full size of the screen and stimuli were spaced 6 s apart. The sequence of 8 directions was presented in a pseudo random order then repeated 5 times. Visual stimulation was synchronized with imaging via Shapeshifter and a U3 LabJack digital-to-analog converter (Labjack, Lakewood, CO, U.S.A.).

Two-photon imaging

Fish were immobilised in 3% low melting point agar (Biogene, Kimbolton, Cambs, UK) with one eye pointing at the middle of the screen. Ocular muscles were paralyzed by injection of α-bungarotoxin around the eye. Bipolar and amacrine cell terminals and RGC dendrites in the central region of the retina were imaged at 10-20 Hz *in vivo* using a Scientifica 2-photon microscope (Scientifica, Uckfield, East Sussex, UK) equipped with a mode-locked Chameleon Ti-sapphire laser tuned to 915 nm (Coherent Inc., Santa Clara, CA, U.S.A.) with an Olympus XLUMPLANFL 20 × water dipping objective (NA 1, Olympus, Tokyo, Japan). Emitted fluorescence was captured through the objective. Scanning and image acquisition were controlled under ScanImage v.3.8 software (Pologruto et al., 2003). Locating the axon terminals of identified RGCs in fish with more than one labeled RGC was aided by the retinotopic distribution of RGC axons in the tectum (Robles et al., 2014) where dorso-temporal RGCs project to the ventro-anterior region of the tectum. We also confirmed that the axon terminal belonged to the identified RGC by laser ablating the RGC to check that the axon terminals stopped responding (Figure S1), this was done by parking the beam over the cell body with the laser power at maximum (800 nm), laser delivery was terminated as fluorescence dramatically increased, this coincided with destruction of the cell body.

Drug application

To administer inhibitory antagonists to the retina *in vivo*, we injected $\sim$4 nL of a solution containing 10 mM gabazine and 10 mM strychnine. We confirmed that these drugs gained access by including 1 mM Alexa 594 in the injection needle, within 5 mins of injection the dye could be detected within the inner plexiform layer of the retina (Figure S5A). However, the drugs do not distribute evenly within the eye, as can be seen in the accumulation of Alexa 594 in the intravitreal space.

QUANTIFICATION AND STATISTICAL ANALYSIS

Image segmentation

Regions of interest (ROIs) corresponding to synapses were segmented from the registered time series using an iterative method, similar to (Portugues et al., 2014). Initially we determined a local correlation map by cross correlating the time series of each pixel (x0) with its 8 neighbors (x1-8), with pixel x0 then being replaced by the maximum correlation value. This local correlation map was then used to seed the ROIs. The pixel with the highest value in the local correlation map provided the first ROI seed. This pixel had 8 nearest neighbors, each was tested for correlation with the seed and if a threshold was reached they were added to the ROI. This process was iterated for the neighbors of all pixels added to the ROI, when no further pixels were added the ROI was complete. The next ROI seed is chosen as the highest value from the remaining pixels in the local correlation map. Threshold values were chosen by the experimenter and were consistent across all fish analyzed for a particular protocol. Only terminals that had responses > 4*SD of the base line were included for subsequent analysis. For segmentation of iGluSNFR signals in the tectum: the same approach was used however care was taken to join ROIs whose time series were strongly correlated. This ensured that we did not attribute the output of one RGC giving rise to multiple synaptic terminals to outputs from multiple RGCs, but may mean that we underestimated the frequency of some responses types if separate cells generated two similar response types.

Classification of retinal ganglion cells

Cells were classified as "dynamic predictive coding" if they responded with a transient on each transition that was greater than the steady state of the previous response by at least 4 standard deviations. RGCs were classified as orientation selective if the responses to the final two gratings differed by 4 standard deviations of their steady-state responses.

Quantifying orientation selectivity

Wherever stated the orientation selectivity index (OSI) was calculated using Equation 1, where a and b are the responses to the last two orthogonal angles thus avoiding any contrast dependent adaptation ("responses" are defined below).

$$OSI = \frac{|a - b|}{|a| + |b|} \qquad \text{(Equation 1)}$$

A response was only considered to have occurred at any given orientation if the signal differed by 4 times the median absolute deviation (MAD) of the preceding 15 s baseline. The value of a response was then determined in the following way: 1st we determined if the response was transient by comparing the maximum value measured within 0.5 s after the transition, if this was > 4*MAD of the median response over the whole 10 s then the maximum value was used as the response, otherwise the response wasn't considered transient and the median value was used. To prevent noise from generating spurious orientation selectivity, synapses were only classified as orientation selective if the responses to orthogonal orientations differed by greater than a multiple of the noise in the steady-state response which was taken as 4*MAD for tectal responses and 3* MAD for bipolar cell synapses.

Equation 1 was used to define OSI for SyGCaMP measurements from bipolar cell in response to gratings. The amplitudes of the latter two grating where compared where "a" was the median amplitude during 10 s at 90° and "b" was the median amplitude during 10 s at 0°. Responses were only assigned an OSI if the difference between a and b was greater than twice the MAD of the smaller response.

Analysis of responses to moving bars

For SyGCaMP6 measurements from BPC and amacrine terminals we classified terminals as orientation-selective by computing a vector sum in orientation space for each of the 10 trials, terminals were then classified as OS based on the result of Moore's version of Rayleigh's test, with the critical value set to give a false positive rate of 1%. This critical value was determined by performing the same analysis on all terminals with the angle information shuffled; we then used the critical value that classified 1% of terminals as OS in this condition. Similar to previous reports in mouse, we did not find any significant direction preference in bipolar cell terminals (Yonehara et al., 2013), so responses to the same orientation were pooled.

Receptive field reconstruction

The receptive fields of bipolar cell terminals were mapped as described previously (Johnston et al., 2014), briefly a −100% contrast bar was flashed onto the retina with 3.2° spacing and at 5 angles ranging from 0° to 144°. Bars were presented in a pseudo random order and for 0.5 s with a 2 s duty cycle. These responses allowed the receptive fields to be accurately recovered with the filtered back projection method (Johnston et al., 2014).

Modeling the contribution of RF ellipticity to OSI

Phenomenological models were constructed in Igor Pro (Wavemetrics, Oregon, U.S.A.). 404 bipolar cell receptive fields were constructed as normalized 2D Gaussians with the major axis, minor axis, and theta determined from fits to the 404 measured receptive fields (Figure 3B). Each model 2D Gaussian was first multiplied by a matrix representing a single grating stimulus, then the sum of all elements from the resulting matrix was rectified to give the response to a single grating. This was then repeated for the orthogonal orientation and Equation 1 was used to calculate the OSI. This was repeated for all the spatial frequencies tested and the results shown in Figure 3D are the mean ± SD for all 404 receptive fields.

Modeling the filter resulting from feedforward inhibition

We used the equations that fit previous measurements of the excitatory and inhibitory conductances from (Johnston and Lagnado, 2015) and placed a single excitatory and inhibitory synapse at the same point of a single dendrite of the simplified NEURON model used in Figure 7B. The conductance of the excitatory synapse was set to 0.0005 nS and the conductance of the inhibitory synapse was varied from 0 to twice the magnitude of the excitatory synapse. To calculate the temporal filter resulting from the feed-forward inhibitory synapse we used normalized-least-mean-squares adaptive filtering with a filter length set to 750 ms. The filter was fit to 804 s of data generated by the simplified NEURON model which contained 1600 randomly timed synaptic events of varying duration.

Neuron, Volume *102*

Supplemental Information

A Retinal Circuit Generating a Dynamic Predictive Code for Oriented Features

Jamie Johnston, Sofie-Helene Seibel, Léa Simone Adele Darnet, Sabine Renninger, Michael Orger, and Leon Lagnado

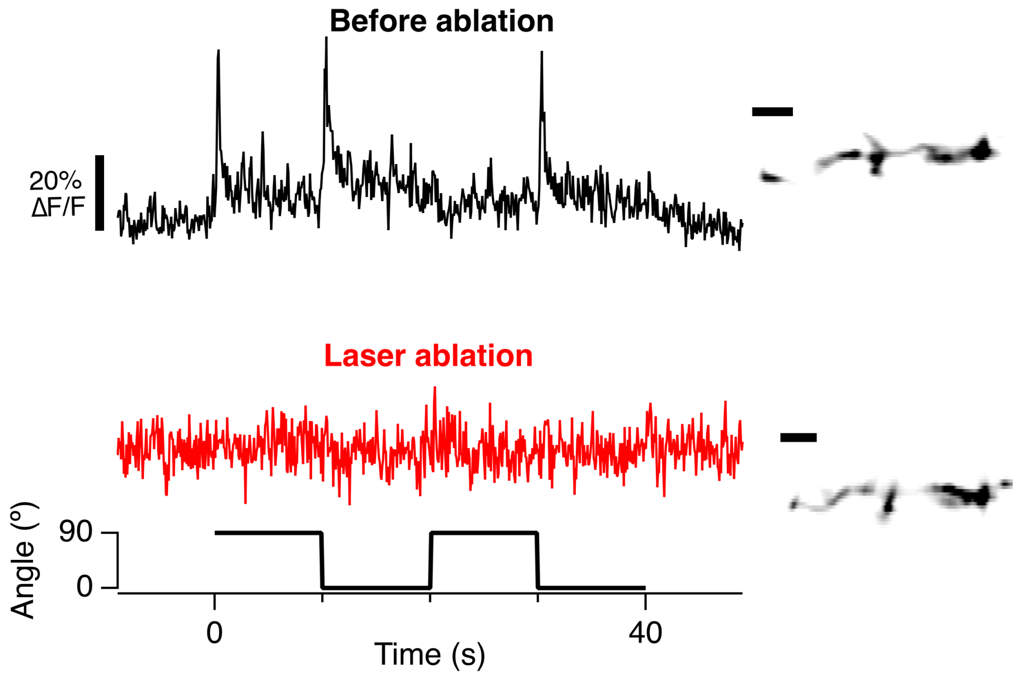

Figure S1. iGluSnFR signals were abolished after laser ablation of the soma, related to Figure 1. The iGluSnFR signal from an RGC axon terminal in response to a full-field grating reversing contrast at 5 Hz that switched orientation from 90º to 0º at 10 s intervals. This RGC was statically tuned to the vertical. The black and red traces show the responses before and after laser ablation of the RGC's cell body. The axonal arborization in the tectum is shown to the right. Scale bar 5 μm. This behaviour was observed in all three RGCs tested in this way, indicating that iGluSnFR signals measured in the axon of RGCs did not reflect glutamate spillover.

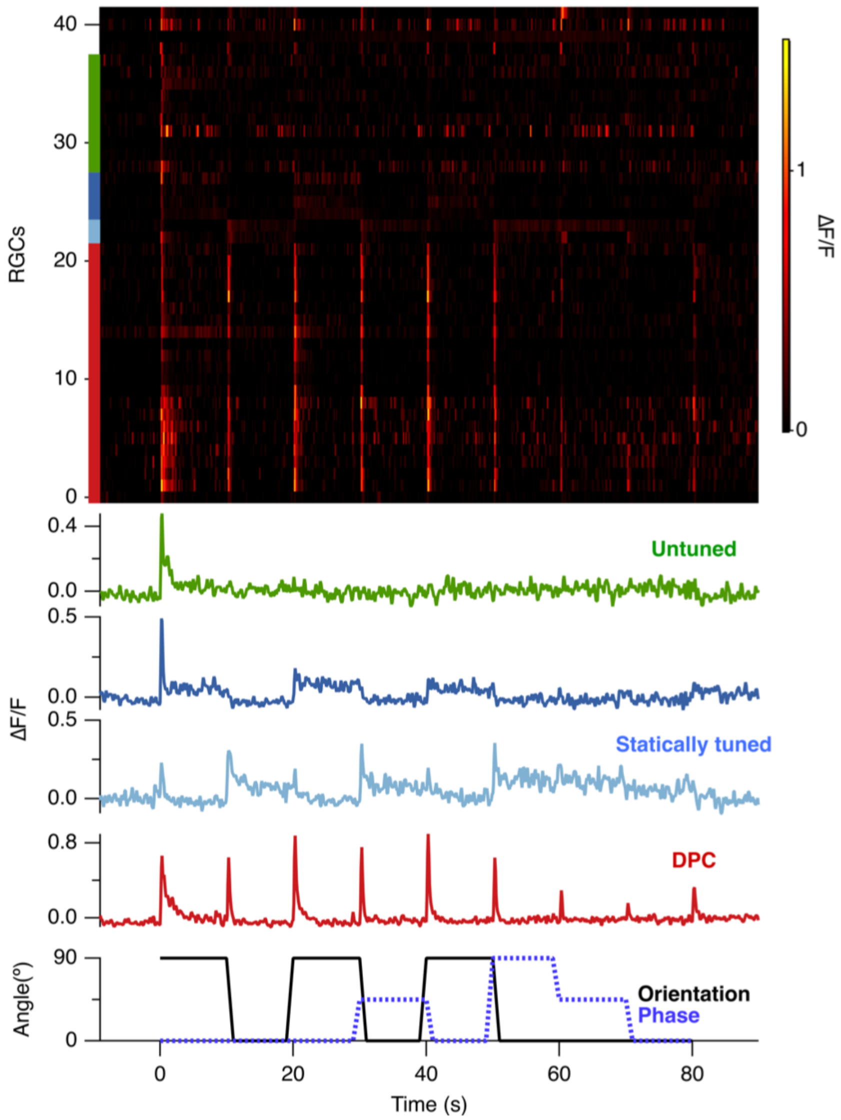

Figure S2. Responses to changes in orientation were not caused by changes in local luminance, related to Figure 1. Raster plot of the responses of 42 retinal ganglion cell terminals to a full-field grating reversing contrast at 5 Hz that switched orientation from 90° to 0° at 10 s intervals. Shifts in orientation were applied at three different phases of the grating (0°, 45° and 90°) and then phase changes alone (90° -> 45° and 45° -> 0°) were applied at a constant orientation of 0°. The traces below are the average responses for neurons classified as either untuned to orientation (green), statically tuned (light and dark blue) or those exhibiting dynamic predictive coding (red). Note that the 22 DPC neurons generated very similar responses to changes in orientation at different phases of the grating and that changes in phase alone generated significantly smaller responses.

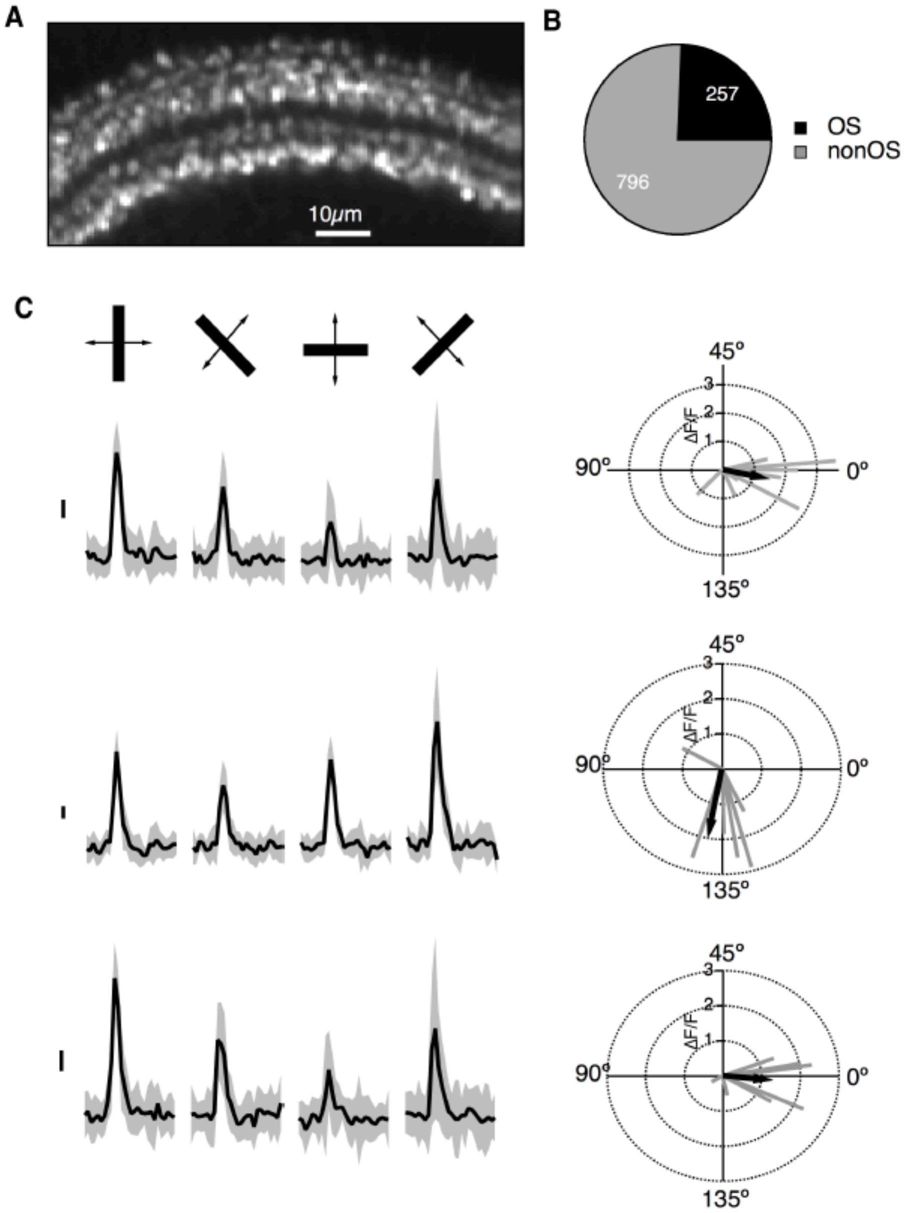

Figure S3. Orientation selectivity in bipolar cell synapses measured with moving bars, related to Figure 3. A) An array of bipolar cell terminals labelled with SyGCaMP6f imaged *in vivo*. **B)** 25% of 1053 measured synapses displayed a significant orientation preference with a false positive rate of 1%. The distribution of orientation preferences for these terminals are shown in Fig. 3F. **C)** *Left:* Example responses from 3 terminals to bars of different orientations moving across the field of view mean (black) ± SD (grey) of 10 repetitions. *Right:* The vector sum of each of the 10 trials plotted in orientation space (grey) with the average vector sum shown in black. To detect whether an individual terminal displayed a significant orientation preference we performed Moore's version of Rayleigh's test on the distribution of vector sums.

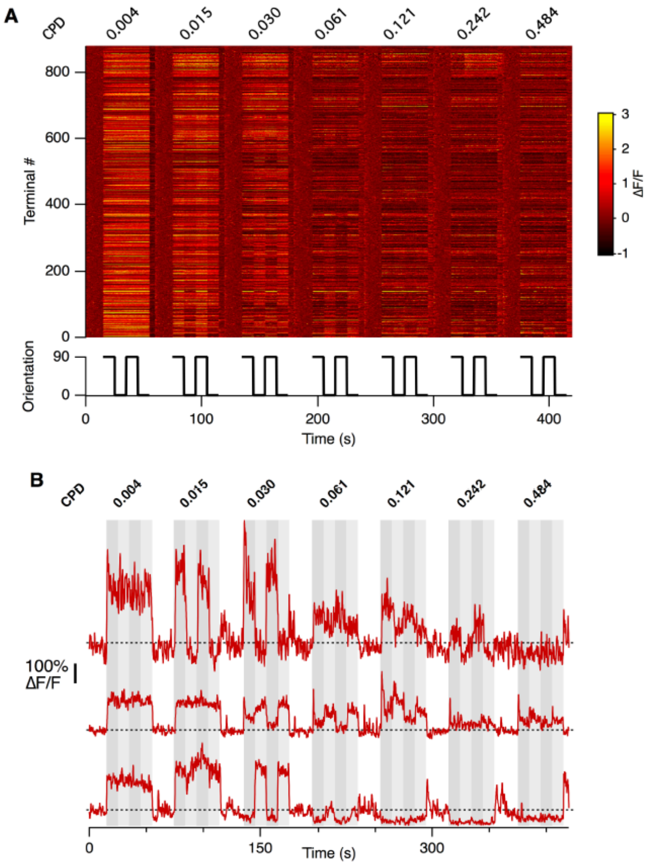

Figure S4. Spatial frequency tuning of bipolar cell synapses measured with SyGCaMP6f, related to Figure 3. A) The responses of 819 retinal bipolar cell synapses to gratings of spatial frequency ranging from 0.004 cycles per degree (CPD) to 0.484 CPD, 0.004 CPD is equivalent to full field. Each grating was given in a pseudo random order and then de-shuffled and concatenated for display purposes. This data generated the spatial frequency tuning shown in Fig. 3D. **B)** Examples from 3 bipolar terminals to gratings of varying spatial frequency.

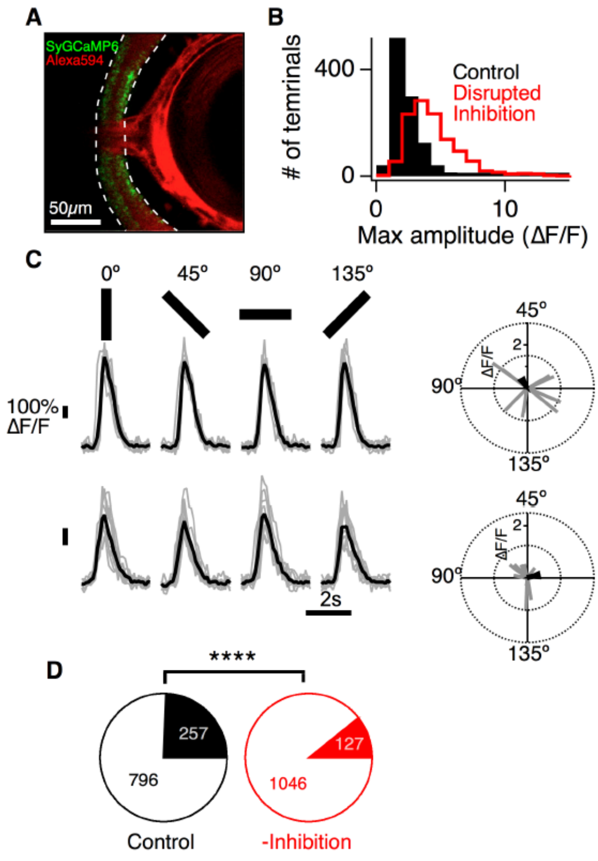

Figure S5. Inhibition contributes to the orientation sensitivity of bipolar cell terminals, related to Figure 3. A) Wide field of view of a zebrafish eye after an intravitreal injection of ~4 nl of a solution containing 10 mM strychnine, 10 mM gabazine and 1 mM Alexa 594. The inner plexiform layer is demarcated with dashed white lines with SyGCaMP6 expressing bipolar cell terminals labelled in green. The Alexa 594 signal is concentrated in the vitreous chamber and can be detected in the inner plexiform layer. **B)** Histograms comparing the average peak amplitudes of the response to the preferred stimuli for control (black, n=1053 terminals) and with intravitreal injection of inhibitory antagonists (red, n=1173, 7 fish). With inhibition blocked the responses of bipolar terminals tended to be larger ($P<0.0001$, Mann-Whitney test). **C)** Example responses from 2 terminals to bars of different orientations moving across the field of view, recorded in a retina with inhibitory antagonists blocked. Grey traces represent the 10 individual trials with the average shown in black. *Right:* The vector sum of each of the 10 trails plotted in orientation space (grey) with the average vector sum shown in black. **D)** The proportion of OS terminals was significantly lower in retinae where inhibition was blocked (red) when compared to control (black), $P<0.0001$, Fisher's exact test).
